# The importance of incorporating systems thinking and One Health in global health classrooms: findings from a One Health simulation activity

**DOI:** 10.3389/fpubh.2024.1299116

**Published:** 2024-02-28

**Authors:** Daniel Acosta, Heather Stark, George Hack

**Affiliations:** ^1^Department of Environmental and Global Health, College of Public Health and Health Professions, University of Florida, Gainesville, FL, United States; ^2^Department of Epidemiology, College of Public Health and Health Professions, University of Florida, Gainesville, FL, United States; ^3^College of Public Health and Health Professions, University of Florida, Gainesville, FL, United States

**Keywords:** pedagogy, public health education, One Health, teaching, education, simulation, undergraduate

## Abstract

There are several challenges and opportunities in health education in global health. Given the field’s rapid expansion, demand for including systems thinking and One Health (a unifying approach that considers human, animal, and environmental health) in global health courses has recently increased. Simulation activities provide an avenue to attain and assess learning objectives that foster critical and systems thinking. This study carried out a One Health simulation activity in an undergraduate global health course, conducted a focus group discussion, and obtained responses from written questionnaires from students who participated in the activity. Data were analyzed using thematic analysis. Results show that the One Health simulation was instrumental for students to understand the complex interactions between different actors and stakeholders in global health systems. The One Health simulation also improved class dynamics, peer-to-peer interactions, and collaborations in the remaining part of the course. The activity helped assess two of the critical thinking learning objectives of the course, and there was some evidence that student agency and confidence may have been improved. Evidence shows that the activity helped students understand the principles of systems thinking and apply them in complex scenarios. Findings support including interactive simulation activities in global health courses to include elements of system science and One Health into classroom activities innovatively and engagingly.

## 1 Introduction

The inclusion of global health in the curricula of future public health and health professionals plays an essential role in increasing the cultural competency of future professionals (1, 2). Given the rapid pace of globalization in the last few decades and events such as the COVID-19 pandemic, the field of global health has been expanding in an accelerated manner. However, there are many barriers to teaching global health, including some of the current exploitative and colonial dynamics in the field (3–5). Some of these barriers, such as global health education usually spotlighting and framing Western health systems as superior (3) and how current systems are often complicit in causing and exacerbating health disparities (5), are detrimental to global health. For most students, these challenges are compounded by a lack of experience or interactions with other health systems outside their home countries. Global health educators have argued that the COVID-19 pandemic could help reshape global health education, advocating for increased multimodal class content, interactive assignments, and interactive tools (5). Several domains in the field of global health have been identified as part of a competency model for global health practitioners, such as the ability to collaborate, form partnerships, political awareness, project management, and strategic analysis (6). Covering such a broad set of competencies while addressing current dynamics in the field can be daunting for educators. Implementing innovative approaches that bring more fidelity into class problems is necessary to foster a richer and more complex engagement with students.

Given our current context of globalization and intricately interconnected systems, alongside the challenges mentioned above, growing arguments have expressed the need for global health education to be accompanied by systems thinking approaches (7). The advocacy for including system thinking approaches in health has been particularly evident in One Health. One Health is an approach that strives for optimal health outcomes, recognizing the interconnections between the environment, people, and animals, and is often a process that involves multisectoral and transdisciplinary collaborations (8). Many practitioners from different fields have argued that systems thinking approaches are critical to One Health practice (9–12). Studying One Health is also very pertinent to global health, as connections between the environment, animals, and humans on a global scale are often different across countries, as the percentage of populations engaged in agriculture usually varies significantly between high-income and low-income countries. The interconnectedness of One Health with various systems (e.g., economics, politics) has generated advocacy among educators to include One Health in early undergraduate education in other fields beyond health and veterinary medicine (13). Successful programs have implemented educational strategies rooted in a One Health framework at a high school level and a Master’s level, which have shown the potential to foster interdisciplinary collaborations (14). Given the growing need of including One Health in curriculum across different disciplines, the development of innovative teaching methods could provide educators with tools to meet the needs of students.

Several pedagogical methods in system science, such as scenario planning, agent-based modeling, and network analysis, are relevant to global health systems (15). However, using these methods as practical teaching tools can be challenging, as they were designed as data collection and analysis tools rather than teaching strategies. Simulations, however, have been used in teaching systems thinking in other disciplines, such as Business and Logistics (16, 17). Logistics and business management educators have utilized simulation-based teaching strategies, such as the Beer Distribution Game, which has shown to be highly effective in fostering systems thinking among undergraduate students in those fields (18, 19). There have been approaches that aim to bridge theory and practice in global health, such as global health case study competitions, which have proven incredibly successful in engaging students and cultivating analytical thinking skills (20). A simulation approach in global health could serve as an effective teaching tool to foster systems thinking, as it has been done in other disciplines.

Driven by the need to include a systems thinking perspective alongside the concept of One Health in a Global Health course for undergraduate students, the research team designed an interactive simulation where students played different roles in a fictional country facing several health threats. Our research question, which is of exploratory nature, is to determine if the use of interactive simulation tools, such as those utilized in other fields, are effective tools in the instruction of One Health in a Global Health course. We hypothesize that a simulation activity rooted in systems science would be an effective tool for attaining the learning objectives of a Global Health course at the undergraduate level. The two learning objectives that the research team wanted to assess were: students should be able to use evidence to assess priority illnesses and threats in different contexts; and students should be able to identify and compare appropriate interventions or solutions for specific health threats in different contexts. Students needed to consider the political, economic, and health consequences of their decisions within the system of government of the fictional country. Our objective was to pilot this educational strategy and assess the feasibility of the approach to foster student achievement of two of the course’s learning objectives. Additionally, we aimed to understand the impact of the educational strategy on student engagement and student perceptions of global health challenges.

## 2 Methods

### 2.1 Action research approach

This study used an action research approach with qualitative data collection methods and analysis. Action research has been widely used in educational settings (21) and in the context of understanding the effects of technology in the classroom (22). Action research is usually conducting in four stages of a cycle: planning, action, observation, and reflections.

#### 2.1.1 Planning

During the planning stage, we identified the current teaching strategies for the One Health module of a Global Health course. This consisted of lectures and short facilitated activities, all which lacked a strong system thinking component. Given the increasing calls to include systems thinking as part of One Health education (9–12), the research team identified the potential use of simulation as a way to incorporate systems thinking in the content of the One Health modules. We then proceeded to establish our research framework, guided by our research question, which is to determine if the use of interactive simulation tools, such as those utilized in other fields, are effective tools in the instruction of One Health in a Global Health course. We then identified the appropriate timeframe to implement the study and data collection methods better suited to answer our research question. A qualitative approach was used as it provided flexibility to explore a broader range of themes about the student’s experience.

#### 2.1.2 Action: the simulation

During the Spring semester of 2023 (January–May), undergraduate students in a required Global Health course at a large public university participated in an interactive One Health simulation designed by the instructor. The activity was carried out halfway through the semester after students had participated in several lectures and completed assignments that related to topics covered in the activity. The previously covered topics include communicable diseases, non-communicable diseases, neglected tropical diseases, global stakeholders, and One Health. Participation in the activity was part of the course and was completed by all students, while participation in the study remained optional. The activity was conducted in three classroom settings, ranging from 44 to 50 students per session.

The simulation consists of a fictional country with a national government, a Non-Governmental Organization (NGO), and multiple state-level government systems. The objective of the simulation is to minimize the number of Disability-Adjusted Life Years (DALYs). Each student assumes the role of a different stakeholder responsible for making decisions about a health crisis in the fictional country. During the simulation, there are several scenarios in each country’s state where different challenges are presented to each group. There are two types of groups in this activity: those at the national level and those at the state level. At the national level, there are two institutions concerned with making decisions for the country from a One Health perspective: the national level government of the country (Prime Minister, Minister of Livestock, Minister of Agriculture, and Minister of Health) and an international NGO. Within these national-level groups, each student has a different role. At the state level, each state has a governmental entity that makes decisions about health in their region and reports these decisions to the national-level organizations. Each state has unique challenges and opportunities. Each state has four students, each with a different role (Mayor, Senior Veterinary Officer, Senior Health Officer, and Senior Agricultural Officer). Based on information provided during the simulation, each group must decide how to tackle these issues using a One Health approach.

**Figure 1:**
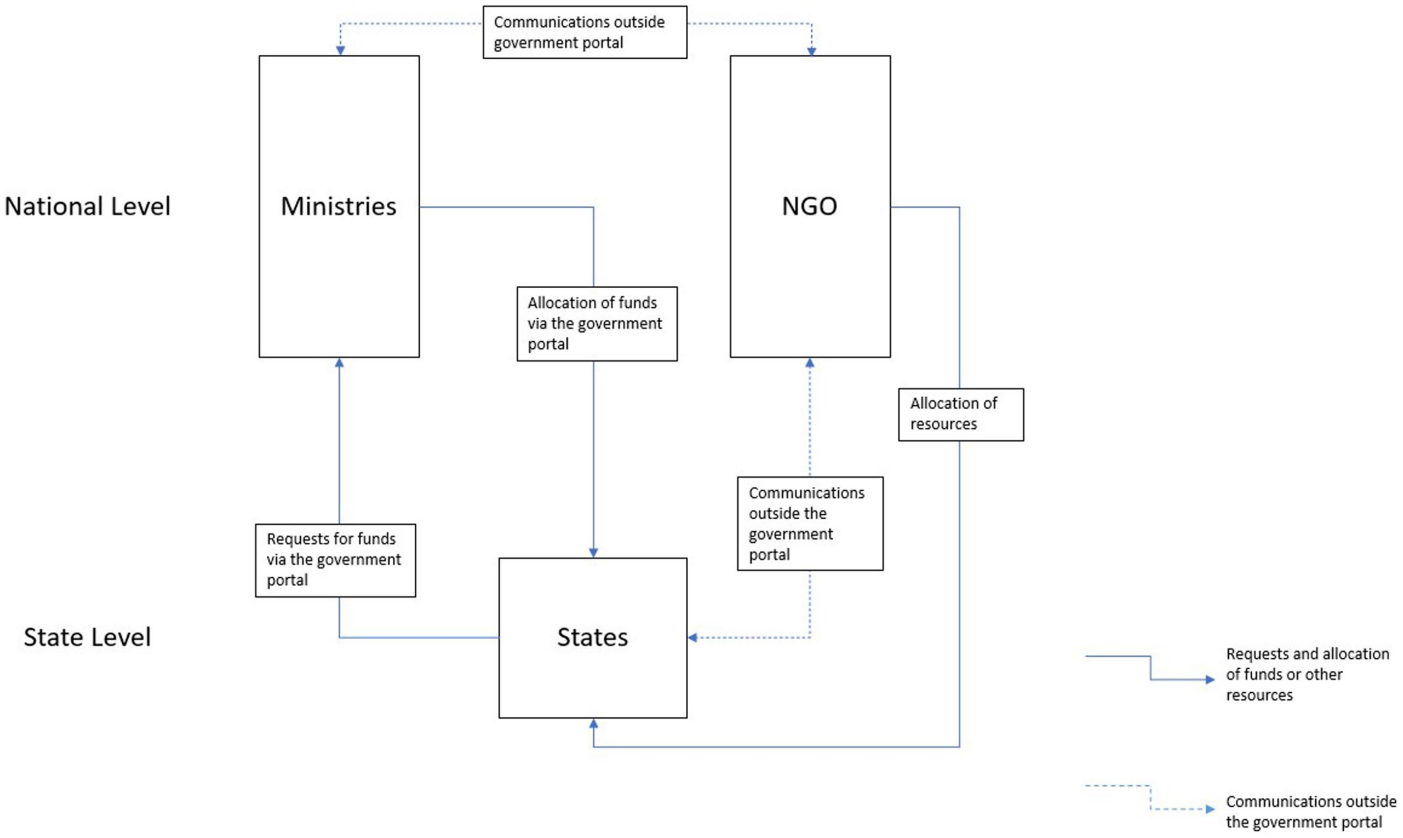
Channels of communication and resources between the national and state levels.

**Figure 2:**
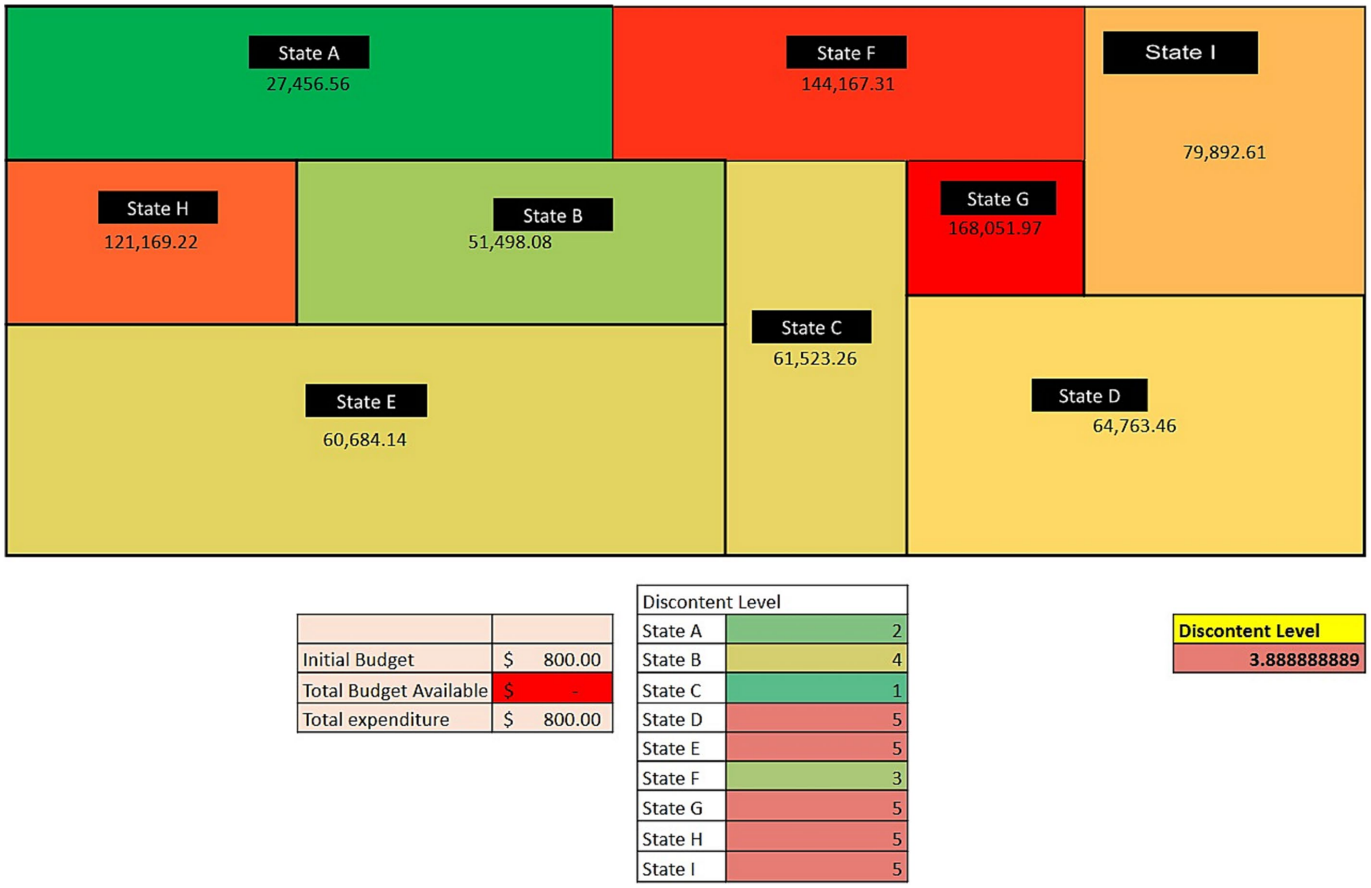
Map of the fictional country with the results of a completed simulation after round three, displaying the DALYs of each state.

All the state-level groups comprised four students, while the national-level groups had 6–8 students, meaning there were 10–11 groups (depending on the classroom size, an additional state was added). Two students could play the same role at the national level (e.g., the Minister of Health was composed of a team of two students within the national level group). A week before the activity, the instructor provided directions for the activity and provided each student with a document describing their group’s current situation. For instance, students in “State A” received a different copy than students from “State B,” and so on. In these documents, the context of the current situations of the states was provided. The document also included a very brief overview of the country. Students in groups at the national level received a different document, which had high-level insights into each state’s inner workings. Students were instructed to keep these documents private from students from other groups. The simulation lasts 2 h, but students must come prepared by reading the short documents provided a week in advance. Figure 1 shows the structure of the government of the fictional country, as well as channels of communication. Communications and requests via the government portal are public, meaning all stakeholders can see them. Communications outside the portal are not public, but actors are free to share these communications as they see fit (e.g., the decision about what states the NGO is going to support). The activity consisted of three rounds. At the start of each round, each state requests funding from the national level government alongside a justification for the requested funds. The national government then allocates funds as they see fit to each state, and the round ends when the states decide how to invest funding and personnel made available to them by the national-level groups. The NGO can provide additional funding and personnel to a limited number of groups. After each round, the interactive map (Figure 2) updates and shows the current cumulative DALYs in each state based on their decisions and how they spent the funds.

During the activity, an interactive Google Sheet was used to simulate the government portal to requisition and spending of funds. After each state allocated funds and personnel to specific activities (e.g., distribution of malaria nets), the number of DALYs each state had accumulated so far got updated automatically. It was reflected on a map projected for all the class to see (Figure 2). The objective of the simulation was to minimize the number of DALYs in the country. However, each state had its own set of interests and challenges. After round 2, when there was only one more round, a series of simultaneous stakeholder meetings took place. In these stakeholder meetings, groups break up disciplinarily (e.g., those acting as Health Officers from each state get together with the Minister of Health). In these meetings, students from different states must strategize the funding priorities in each sector (health, livestock, agriculture). After the meetings, students returned to their groups, where they debriefed the outcomes of the meetings and strategized for the last round. Once the last round was finalized, each group could see the number of DALYs accumulated throughout the simulation based on their decisions. The set of instructions and scenarios utilized in the simulation can be found in the Supplementary files.

#### 2.1.3 Observation: data collection and analysis

Qualitative data was collected from students using focus group discussions and a questionnaire. A focus group discussion was conducted with volunteers from the three different sections of the course. Focus Group Discussions allow researchers to obtain rich qualitative data as it enables the expression of a broad range of ideas and views from different participants (23). Six students participated in the focus group discussion, which was conducted by a researcher who was not one of the instructors of the course. The instructors of the course were absent during the focus group discussion. Participation in the focus group discussion was optional, and no compensation was offered for student participation. The focus group discussion took place 4 weeks after the activity was completed. The recording was de-identified and transcribed for analysis. Students in the focus group were asked to express their overall thoughts about the simulation, and then probed into highlighting what they identified as weaknesses and strengths. The facilitator also asked the students to share what they had learned from the activity, which was used to assess to what degree had the simulation contributed to the student’s learning objectives. Feedback on the students’ experiences in the activity was also sought by the facilitator to identify potential points of improvement in future iterations of the activity.

The other data source used was a written questionnaire students answered about the activity, where they could decide to opt in or out of the study, with this decision bearing no influence on their grades. These questions probed deeper into students’ perceptions of how they viewed different of One Health interventions at the global level. The written questionnaire had general questions about their main takeaways from the simulation, as well as other more specific questions that aimed to evaluate students’ attainment of systems thinking in One Health. To evaluate this, students were asked to express their opinions on the role of institutions at different administrative levels, as well as how their horizontal and vertical interactions played a role in carrying out One Health interventions (based on their experience in the simulation). Data from the focus group discussion and the written questionnaires were analyzed using inductive thematic analysis (24). The transcription from the focus group and the written answers from the questionnaires were analyzed in parallel using an inductive approach, in which analysis was carried out without preconceived themes. After an initial reading the transcript of the focus group and the responses of the written questionnaires, the researchers coded the results after identifying three main research themes. This study protocol was exempted by the University of Florida’s Internal Review Board and consent was obtained from all participating students.

#### 2.1.4 Reflections

The analysis of the results and findings will be used to determine whether the simulation activity is an effective teaching tool at the undergraduate level to facilitate learning outcomes and embed systems thinking into One Health modules. Revisions to the simulation activity might also be carried out if the data suggests that there are weaknesses or potential avenues for improvement. The reflection stage of our action research plan will serve to inform whether the activity will continue to be carried out and potentially expanded to graduate-level courses.

## 3 Results

Three main themes were identified in the data obtained from the focus group discussion and the written questionnaires. Students highlighted the effectiveness of the simulation in improving educational strategies in global health, gaining a better understanding of health systems, increasing a sense of agency, increasing confidence in decision-making, improving the class environment, and fostering peer-to-peer collaborations.

### 3.1 Effectiveness as a teaching tool

Several participants in the focus group discussion and the written response emphasized the effectiveness of using an interactive strategy as a teaching tool. One of the main recurring themes was how the activity allowed for a better understanding of the complexity of global health beyond what other teaching methods, such as lectures or videos, could accomplish. The simulation activity also effectively assessed students’ progress toward the course’s learning objectives. In most cases, students could correctly prioritize issues in the simulation based on the information provided using a One Health framework. Students could also compare different interventions and proposed solutions to address a health threat in the specific context of the simulation. Most importantly, students could take a system thinking approach when strategizing their following actions during the simulation. There were several instances where students reported having discussions about possible scenarios of different strategies and how these scenarios served to inform their decisions moving forward. This shows how students could utilize elements from systems science methodologies to address the issues posted in the simulation. There was consensus among the students that the activity should remain a part of the course. Participants also mentioned that they would have liked to repeat the activity, playing another role in deepening their understanding of the system. The following are student quotes when asked about the activity as a teaching tool in the class context.

*“Ultimately, doing simulations like this, it really does stick in our minds. We did something different that sticks in my mind, so I remember that. Now I feel more connected to the professor and students around me. I honestly feel that that has been missing from the classroom, taking away the power from the professor and giving it to the students. In that, the students will actually care more.”*

*“One thing I liked about this activity is the freedom we got to make our decisions and learn from the mistakes.”*

*“I really enjoyed the way that we were all able to interact like actual public health professionals. And, I got to hear what other people expertise and knowledge and beliefs and opinions were, and see how those interacted with mine, and how you could combine our efforts to the overall goal of reducing the DALY’s.”*

### 3.2 Understanding system interactions in the context of One Health

A recurring theme in the focus group discussion and the written responses was that the activity allowed students to comprehend beyond theoretical frameworks how health is interconnected with other disciplines, creating complex interactions. Students highlighted that this activity allowed them to understand how interconnected health and sectors such as agriculture and livestock production are. There were also mentions of how the simulation allowed for a more nuanced understanding of how global health challenges do not occur isolated from each other, and these combinations of health threats prove to be more challenging to address than a single issue. Students also commented that the simulation showed how power dynamics could have critical implications on a system, specifically on health issues, as they relate to other areas such, as the economy. The following are quotes from students when asked about the main takeaways from the activity.

*“I feel like when you put a lot of separate concepts throughout the course, and by themselves they seem like pretty easy, but when you like put them, all together like in a singular situation, it shows how one can affect the other and that’s why it’s so difficult.”*

*“In my classes thus far, lecture has been the focal point. This activity was a hands-on way of learning, which is typically how I learn best. It also made learning about public health more fun and engaging, furthering our knowledge on the topic. By having this hands-on experience, it did help me understand the complexities of global health.”*

*“The activity extended the course material on One Health, international NGOs, and national governments that textbooks and videos could not compare to.”*

*“During the assignment the officers in my state and I were constantly making decisions to ensure that the economic stability of our country would remain intact. This assignment just gives me a glimpse of all the outside factors that can affect health decisions. I think the biggest thing I learned from this activity is how difficult it is to make health decisions when juggling outside factors.”*

### 3.3 Student agency and class environment

Students also expressed their enthusiasm about engaging in an activity where they felt they had freedom of decision to act as public health professionals. This theme emerged in the focus group discussion and in the written responses, where students alluded to how the simulation granted them a space to test their knowledge with the freedom to express their ideas and even make mistakes without real-life negative consequences. This could show that the One Health simulation might improve student agency and confidence. The grading rubric of the activity was not tied to the performance of each group during the simulation. Instead, it was linked to an analytical paper where students reflected on their performance in the activity. This seemed an effective way to remove pressure from students, and some mentioned this fostered an environment where they felt comfortable thinking “outside the box” without risking a negative grade.

Another theme that emerged from the data alluded to how the activity changed class dynamics moving forward in the semester. Students underscored the effectiveness of the simulation in fostering an environment of group work collaborations with their peers, contrasting it with other group projects in which collaborations are often limited to the division of tasks. The simulation activity was designed to enable the students to use a system thinking approach and the application of a One Health framework, however, it was also able to foster better relationships in the classroom between students, their peers, and the instructors. Activities such as the simulation can help create a better class environment by increasing meaningful interactions between students.

*“So that whole interaction facilitated more conversation than other group work where you just separate [tasks] and do not really interact. At least in this activity we were all interacting because we all had issues that we needed to solve and talk out and make sure that we were tackling them in the best way possible. This definitely facilitated conversations between peers.”*

*“The way that the activity was set up in that the problems all interact with each other, it forces you to have those conversations. Because, this issue is not only impacting agriculture, it will impact [the] health of citizens, so you need to converse with the health official to discuss that, and how your decision is going to change that.*

*“Engagement in classes are fun, and these interactive activities are really fun. Because it does give you the opportunity to interact with your peers more.”*

## 4 Discussion

The results show that using simulation-based active learning strategies in the context of global health provides several benefits to the classroom. We also found the simulation to be an effective education tool for One Health topics for students in Global Health courses. Embedding system science elements into the educational strategy (in this case the simulation) seemed to have been an appropriate mechanism to attain the desired learning objectives. Students identified the activity as better suited to enable their understanding of the complexities of global health when compared to other teaching methods such as lectures and video recordings. Other studies have shown that games and simulations allow students in other disciplines (e.g., business management) to train themselves in decision-making (25), an important aspect crucial to global health practitioners. Evidence also shows that simulations are better suited to provide students with complex decision-making skills that might be harder to convey using traditional teaching methods (26). Enabling students to practice decision-making in global health, where there are no consequences for others, allows future professionals to obtain some experience in managing complex situations without the pressure of potential adverse outcomes. The One Health simulation might increase student self-efficacy in decision-making in the context of global health, as other studies in different disciplines have found that simulation activities increase student self-efficacy (27–29); however, measuring self-efficacy was not in the scope of the research and should be further explored.

There is also evidence that simulations can improve teamwork and other essential skills, such as social and emotional skills (30). Our findings suggest that the One Health simulation activity nurtured social and emotional skills and other affective outcomes, such as student engagement. After the simulation, students reported improved class dynamics and motivations, which is in line with what other studies have found, where the use of simulations has been shown to advance student engagement (31, 32). Results also suggest that the simulation fostered an environment for effective group work and peer-to-peer communication, essential elements of systems science and global health. The simulation played a role in improving group work dynamics and collaborations, as other studies have shown (33), however, some studies have found conflicting evidence on whether simulations facilitate teamwork or not (34). The One Health simulation was purposively designed to require students to work collaboratively, which could have contributed to the learning process. Evidence shows that peer collaboration is a vital component of the learning process that could be enhanced through games and simulations (35, 36).

Overall, the simulation activity helps foster a better class environment, improve student engagement, evaluate the course’s learning outcomes, enable peer collaborations, engage students in systems thinking approaches, and facilitate discussions about One Health. Based on the results from this study and what researchers observed in the classroom, we recommend that public health educators promote interactive simulations with undergraduate students and potentially with graduate students. Simulations, such as the one piloted in this study, have been widely and commonly used in recent years in other disciplines, such as engineering, economics, business, and logistics (37–39). Public health education should create or adapt these existing teaching tools to enhance the classroom environment and learning outcomes of future public health professionals and practitioners.

There are some limitations to this study. First, the simulation was only piloted with a cohort of undergraduate students in a large research University in the Spring of 2023. Replications with different cohorts of students or students at higher levels (e.g., master’s or Ph.D.) might yield different results. Furthermore, most of the focus group discussion participants were women (5 out of 6), which might reflect the course where most of the students enrolled were women. This does not allow for a gender analysis to determine whether the simulation activity might have been perceived differently in a classroom with a different gender distribution.

## 5 Conclusion

Utilizing a One Health simulation activity can help students understand the complex interactions of different actors and stakeholders in health systems and health outcomes. Simulation activities also increase student engagement and collaboration, increasing the likelihood of achieving the desired learning outcomes. There is some evidence that student agency and confidence could be improved using the One Health simulation. Current curricula in health sciences, and perhaps in other related fields, should include global health, One Health, and systems science early on at the undergraduate level. The use of simulation activities has the potential to improve learning outcomes and provide an avenue for educators to introduce students to complex topics engagingly and fruitfully.

## Data availability statement

The raw data supporting the conclusions of this article will be made available by the authors, without undue reservation.

## Ethics statement

The requirement of ethical approval was waived by University of Florida Institutional Review Board for the studies involving humans because the study was considered exempt, as no sensitive or protected data were collected. The studies were conducted in accordance with the local legislation and institutional requirements. The ethics committee/institutional review board also waived the requirement of written informed consent for participation from the participants or the participants’ legal guardians/next of kin because participants in the focus group discussion were able to consent verbally.

## Author contributions

DA: Conceptualization, Data curation, Formal analysis, Investigation, Methodology, Writing – original draft, Writing – review & editing. HS: Conceptualization, Methodology, Supervision, Writing – review & editing. GH: Conceptualization, Funding acquisition, Methodology, Writing – review & editing.
