# Supplementary material for: The importance of incorporating systems thinking and One Health in global health classrooms: findings from a One Health simulation activity
Source: Front Public Health. 2024 Feb 28;12:1299116. doi: 10.3389/fpubh.2024.1299116 (PMC10933002; doi:10.3389/fpubh.2024.1299116)
Supplement: Supplementary file 2 [file Data_Sheet_1.PDF]

Supplemental Files

Navigating this document

In this document, there is general information about the activity, such as the materials given to students and the rules of the simulation. There are also detailed instructions for instructors who wish to pilot this activity.

Contents

Navigating this document 1
Activity Documents 2
  Scenario 2
    General Overview 2
    Objective 2
    Rules 2
  Actors 3
    National Level: 3
    Sub-national level 4
Instructions for Instructors 5
  1-2 weeks before the activity begins. 5
  Preparation for the Simulation 5
  Familiarizing with the Google Sheet 6
    Student Interfaces 6
    Back-end 9
    Interactive Map 17
  Carrying out the Simulation 18
    Timing 19
    Stakeholder Meeting 19
Context and Details of each State 19

Activity Documents

Scenario

General Overview

The *Republic of Gators* is a country located in the continent of Pangea, in a tropical region with different agroecological zones and it is considered a low-income country (LIC). It's main economic activity is agriculture, with over 50% of the population living in rural areas. The government structure has been historically centralized, but there have been efforts pushing towards de-centralization. This has resulted in a state where major decisions are still made at the national level (strategies, policies, budget, and resource allocation), with some level of independence at the state level.

The following table offers descriptive information about each state:

State	Population (millions)	% pop under poverty line	Stunting rates	Main Agricultural activity
A	0.5	30%	21%	Coffee (for export)
B	2.1	35%	24%	Sugar
C	1.1	60%	34%	Maize
D	1.4	34%	23%	Cattle (farm based)
E	0.8	21%	19%	Tea (export)
F	1.6	45%	41%	Livestock (agro-pastoral)
G	2.1	48%	40%	Sorghum
H	1.5	39%	35%	Livestock (pastoral)
I	0.9	29%	35%	Maize

Scenario: Normally, the Republic of Gators would define a set budget for the year for every sector and district. Given recent climate variability, this year, they have decided to take a more flexible approach with a specialized fund to respond to shocks caused by climate change. These funds are intended to be used only in the following sectors: health, livestock, and agriculture. Resources are pooled together and allocated at the discretion of the Prime Minister. There is a total of $800 Million USD in these funds for the next 3 months. Each state can send proposal on how they envision using these funds. While the national level actors only need to worry about funding issues (and how to allocate funding to each state), each state has unique issues that they need to handle with the resources they have and receive. These issues often are personnel shortages. The rainy season is about to start, and significant parts of the country are expected to see a rise in Malaria. There are also risks for cholera outbreaks, increased schistosomiasis cases, and dengue (new to the country). There are also areas with high risk of livestock disease outbreaks, (e.g., peste des petites ruminants (PPR) affecting goats, and foot and mouth disease (FMD) affecting cattle). There are also concerns in the crop production side, with several pests threatening yields, as well as erratic rain patterns threatening to flood certain areas.

Objective

- Allocate the resources available to the best of your abilities to reduce Disability Adjusted Life Years (DALYs).

Rules

- Each team has 15 minutes to read their specific scenario and strategize within their unit.

- At month 0, each team at the state level will initiate a request for resources to the Prime Minister's (PM) Office, where the Ministry of Health, the Ministry of Crop Production, and the Ministry of Livestock meet.
- The Prime Ministry, and the other Ministries will now decide how to allocate resources to each state.
- An international NGO called INGO operates in all the states and could help alleviate gaps not covered by the government, however, they also have some constraints in resources and what they can provide.
- We will assume that resources reach each state automatically.
- Each state will request a total amount from the PM and will now allocate the resources they got within their unit as they see fit.
- Interventions can be partially funded if the state does not receive the appropriate amount of funds. The closer to the objective, the better in terms of mitigating the impact.
- After resources are allocated, the dashboard will update.
- Each month, a new requisition could be made to the national level actors and resources could be re-allocated.
- The scenario will run for a total of 3 months. The first month will have a duration of 10 minutes. The second month will have a duration of 25 minutes, as there will be a stakeholder meeting (see details below). The last month will last 10 minutes.
- **Stakeholder Meeting:** After Month 1 resources are allocated and DALY's calculated, there will be a stakeholder meeting with a duration of 15 minutes. The Prime Minister will meet with the major's of each state, the Minister of Health will meet with the Health officers of each state, the Minister of Crop production will meet with the agricultural officers of each state, and the Minister of Livestock will meet with the veterinary officers of each state. Members from INGO are invited to participate as well (they can join any meeting). During this meeting, each actor will make a case for securing more funds for their state.
- Elections are coming up soon and the Prime Minister is seeking reelection. If your state is wealthy, or has a high population, you could use this information to your advantage to try and secure more funding.
- If the level of discontent rises above 3.8 at the national level, 200 Million USD of total budget will be lost due to the loss of donor support.

Actors

National Level:

- Prime Minister Office (National Level, 4-8 students)
    - Prime Minister (1-2 student/s)
        - Ministry of Health (1-2 student)
        - Ministry of Crop Production (1-2 student)
        - Ministry of Livestock (1-2 student)

- International NGO "INGO" (National Level, 6-8 students)
    - Country director (1-2 student) *If 0, then decisions are made by votes of the regional directors.

- Regional level INGO director for A, B and C (1-2 student(s))
- Regional level INGO director for D, E and F (1-2 student(s))
- Regional level INGO director for G, H, I (1-2 student(s))

Sub-national level

- State A (Sub-national Level) (4 students)
  - Mayor from State A
  - Senior Veterinary Officer State A
  - Senior Agricultural Officer State A
  - Senior Health Officer A

- State B (Sub-national Level) (4 students)
  - Mayor from State B
  - Senior Veterinary Officer State B
  - Senior Agricultural Officer State B
  - Senior Health Officer B

- State C (Sub-national Level) (4 students)
  - Mayor from State C (Sub-national Level)
  - Senior Veterinary Officer State C
  - Senior Agricultural Officer State C
  - Senior Health Officer C

- State D (Sub-national Level) (4 students)
  - Mayor from State D (Sub-national Level)
  - Senior Veterinary Officer State D
  - Senior Agricultural Officer State D
  - Senior Health Officer D

- State E (Sub-national Level) (4 students)
  - Mayor from State E (Sub-national Level)
  - Senior Veterinary Officer State E
  - Senior Agricultural Officer State E
  - Senior Health Officer E

- State F (Sub-national Level) (4 students)
  - Mayor from State F (Sub-national Level)
  - Senior Veterinary Officer State F
  - Senior Agricultural Officer State F

    - Senior Health Officer F

- State G (Sub-national Level) (4 students)
    - Mayor from State G
    - Senior Veterinary Officer State G
    - Senior Agricultural Officer State G
    - Senior Health Officer G

- State H (Sub-national Level) (4 students)
    - Mayor from State H
    - Senior Veterinary Officer State H
    - Senior Agricultural Officer State H
    - Senior Health Officer H

- State I (Sub-national Level) (4 students)
    - Mayor from State I
    - Senior Veterinary Officer State I
    - Senior Agricultural Officer State I
    - Senior Health Officer I

Instructions for Instructors

In this document, the Excel file, and the other PDF documents found in this supplemental file, you will find the necessary information to run the One Health Simulation.

1-2 weeks before the activity begins.

Before providing students with any details, either assign them to groups or let them for their own groups (group composition should match that in the Actors section). One students have formed their groups, provide all the students with the Scenario (see section with Activity Documents). Walk them through the rules and answer any questions students might have. Provide each group with their specific actor document. For instance, provide those in State A, the document labeled "State A Context". The context documents can be found in the supplemental files. Inform the students they are not to share details of their group with other groups until after the activity is over.

Preparation for the Simulation

Upload the Excel file named "One Health Simulation" to Google Sheets. Ask one student in each group to volunteer to manage the "portal." Only grant access to the Google Sheet document to those students who signed up. Those students will need to bring a laptop on the day of the activity. Furthermore, be

sure to password-protect the following tabs in the Google Sheet file: Master Sheet, Feedback Sheet, and Interactive Map. Only the facilitator of the activity should be able to manage and edit these tabs.

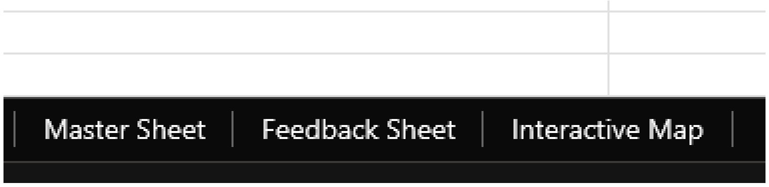

Figure 1 Sheets that need to be password protected

To password-protect these sheets, you can follow these instructions: https://support.google.com/docs/answer/1218656?hl=en&co=GENIE.Platform%3DDesktop

The classroom where the simulation will take place should have at least 1 projector. On the projector, open the "Interactive Map" sheet. All students should always be able to see the map. One of the facilitators will need unlimited access to the Google Sheet, separate from what is currently set up in the projector. At least 2 facilitators are needed to run the simulation. The facilitator should keep time and be able to answer any student questions as they arise, meaning they should be familiarized with the simulation and the Google Sheet. One of the facilitators will be in charge of managing the Google Sheets, which requires full attention for the duration of the simulation.

Familiarizing with the Google Sheet

The Google Sheet is color-coded for ease of use. Cells in orange should not be modified, as they are formulas. Cells in Green should be modified by either the students or the facilitator, depending on the sheet. Yellow Cells are names of important tables that will be referenced in this document. This Google Sheet document has 3 main types of sheets: student interfaces, back-end, and interactive map sheets.

Student Interfaces

These Sheets are what students will see and interact with. There are two types of student interfaces: the Prime Minister Offices sheet and the State's sheet.

State Sheets

These are the sheets students use to request and spend funds at the State Level. It's also where students can express their level of discontent. It's important to highlight that ALL numbers are in millions. Meaning, if requesting 10 million, a student must only type "10". Typing anything different, such as "10M" or "10,000,000" will interfere with the simulation. Decimal points are allowed; typing "10.5" will be interpreted as ten million and five hundred thousand.

Step 1:

The image below shows the top part of the State Sheets. In the green shaded cells of the table called "Amount Requested", students must type in the amount of funding needed for Month 1 for each sector**. Students only need to type a 1–2-digit numbers, the amount typed will be assumed to be in millions.** If a student types "10", this means 10 million. The total is calculated automatically, so if students request 10 million for livestock, 20 for agriculture, and 10 for Health, the Total will be 40, and should be calculated automatically.

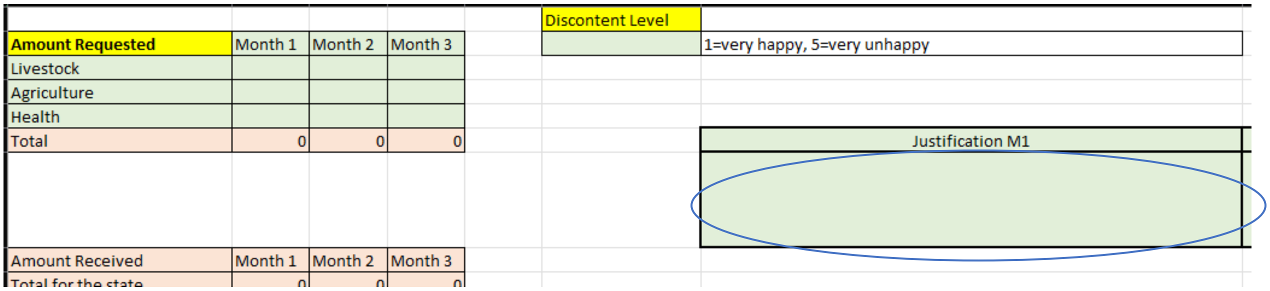

Figure 2 State Level Sheet for Step 1

After typing in the amounts required, students must justify why they are requesting this amount. This will help the national level group to make decisions to allocate funds. Advise students to be concise, if the text is too long, students at the national level will have a hard time reading it. The text should not exceed the length of the box below the one labeled “Justification M1”, which has a blue oval in the figure above. With this, the first step of the simulation, at the state level, is complete.

Step 2:

Once the national level completes allocation, students at each state will be able to see the amount of funds they were allocated (see cells shaded in Orange labeled “Amount Received”, with a blue circle). This is the total amount in Millions received by the state at Month 1. This amount will auto-populate once the national level has allocated funds (see PM Sheets for more details). Now, students must decide how they will use the amount that was allocated. They must type in the number of what they wish to allocate for each sector. This is done in the “Amount Allocated” table for each specific sector and each month (see black oval for month 1). Afterward, students must decide what interventions they will carry out with the provided funds. This is done by allocating personnel. To do this, students must pick one of the two options for each sector in the dropdown menu (see yellow oval on Figure 3). Once this is done, step 2 is completed. At this point, States can also express their level of content/discontent, by selecting a number from the dropdown menu of the “Discontent Level” table. Students can change this at any time.

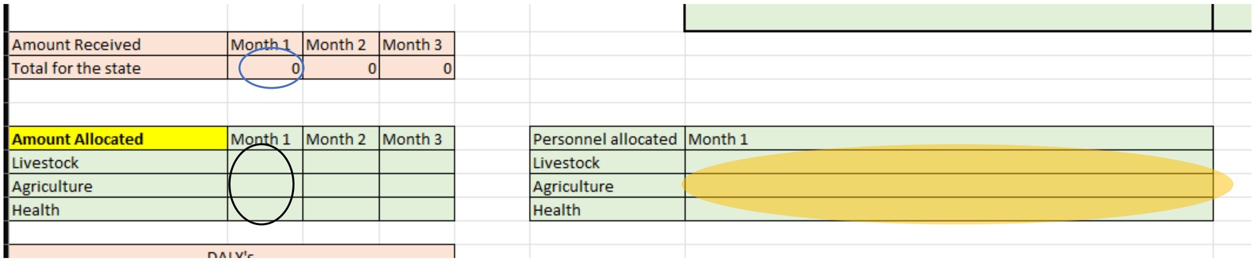

Figure 3 State Level Sheet for Step 2

Step 3:

After the interactive map updates, students will be able to see what the financial needs of their state for the next month are, as well as see how much each sector contributed to the DALY count. Using this information, students should repeat steps 1 and 2, in the areas labeled Month 2 (or M2). Then repeat for Month 3.

DALY's			
	Month 1	Month 2	Month 3
Livestock	0	0	0
Agriculture	0	0	0
Health	0	0	0
Total DALYs	0	0	0
Accumulated DALYs	1500		

Financial Needs	Month 2	Month 3
PPR control needs	0	0
NCD control needs	0	0
Large farm needs	0	0
Small Farm Needs	0	0
Bednet needs	0	0
Fungicide treatment needs	0	0

Figure 4 State-level Sheet for Step 3

PM Sheets

Step 1:

The office of the PM has a sheet labeled "Office of the PM". Here, there are three identical tables, one for each month. Once students at the state level complete step 1, the cell called "justifications" and "amount requested" for each state will populate. With this information, students must allocate funds to each state. Funds are not infinite, and the total of funds given cannot be over what the "Initial Budget" amount is. The initial budget is for the "three months" of the simulation. Figure 6 shows the "Initial Budget". As the PM office allocates funds (by typing the amount in the green cells of the table for each state), the cell called "Total Budget Available" will update; this will allow the office of the PM to assess how many funds are available. Students can try different number combinations before "submitting" the funds to the states.

Step 2:

Repeat Step 1, but in the other table labeled "Fund Request/Allocations Month 2". Then repeat the same process for Month 3.

Fund Request/Allocations Month 1			
States	Justifications	Amount Requested	Amount Given
A	0	0	
B	0	0	
C	0	0	
D	0	0	
E	0	0	
F	0	0	
G	0	0	
H	0	0	
I	0	0	
		Total	0

Figure 5 Step 1 Table for PM Sheet

	in Million of USD
Initial Budget	$ 700.00
Total Budget Available	$ 700.00
Total expenditure	0

Figure 6 PM Budget Table

Back-end

These types of sheets are to ensure that the simulation runs smoothly, and to shield the simulation from student errors that interfere with formulas (e.g., typing “10 million” instead of “10”). This also allows students at the national level to “test” funding strategies before committing to them. At the backend, specifically at the sheet labeled “Feedback Sheet” the facilitator has to complete certain steps in order for the simulation to flow. The diagram below shows the flow of the simulation. In green are the ones where the facilitator has to intervene in order for the simulation to flow.

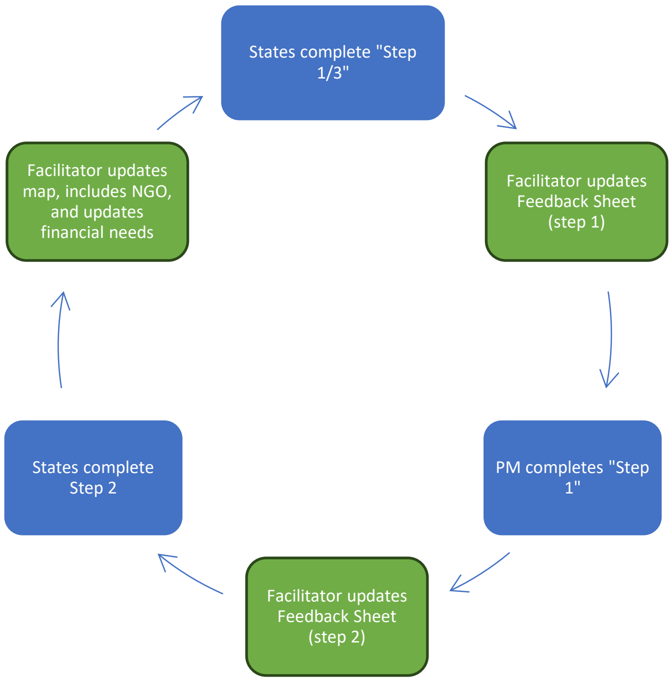

Step 1 for Feedback Sheet:

In this step, the facilitator in charge of managing Google sheets will transmit the information from the state level sheets to the national level sheets. To do so, there are two things they must do.

1. Copy the justifications from the orange cells labeled justifications “Feedback sheet” (see image below) and paste them as text in the green cells also labeled justifications (see figure 7).
2. In the table called Funding Requested M1 in the Feedback Sheet (Cells W31:W40), verify that all the numbers are typed correctly (e.g., 5 or 10). There should be no text in these cells, and there should also be no numbers expressed in millions (e.g., 10,000,000 would be the INCORRECT way to represent 10 million. The correct way is simply: 10). If there are mistakes, tell the students from the states with mistakes to amend the error. Once you have completed this, copy the numbers from the orange cells and paste them below, as shown in Figure 8.

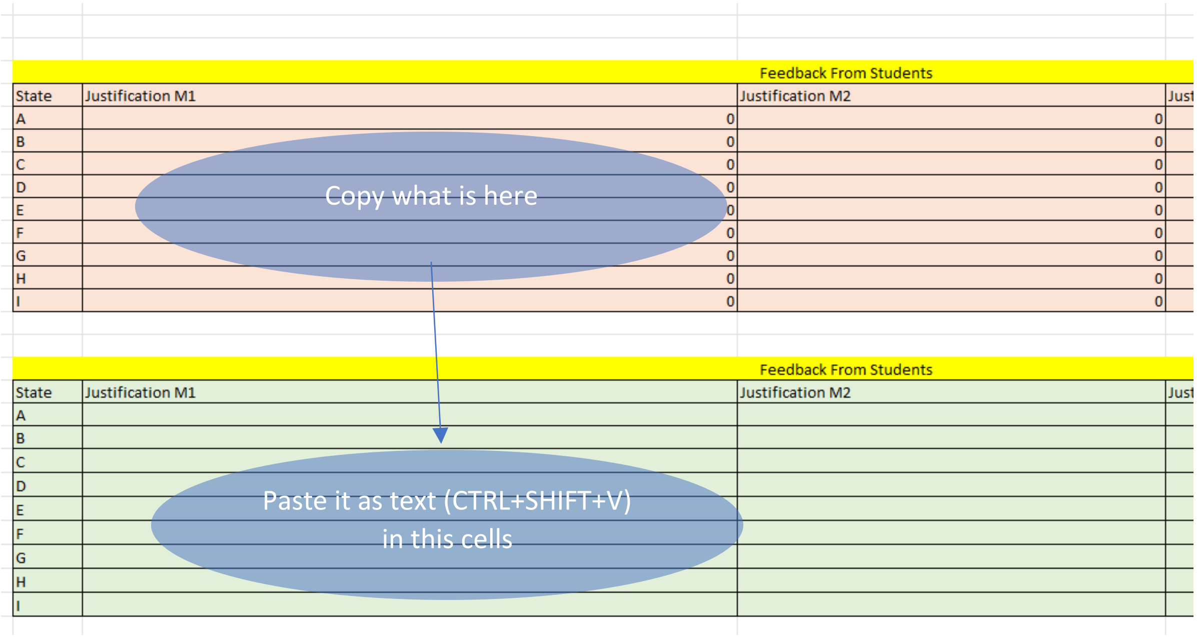

Figure 7 Step 1.1 Feedback Sheet

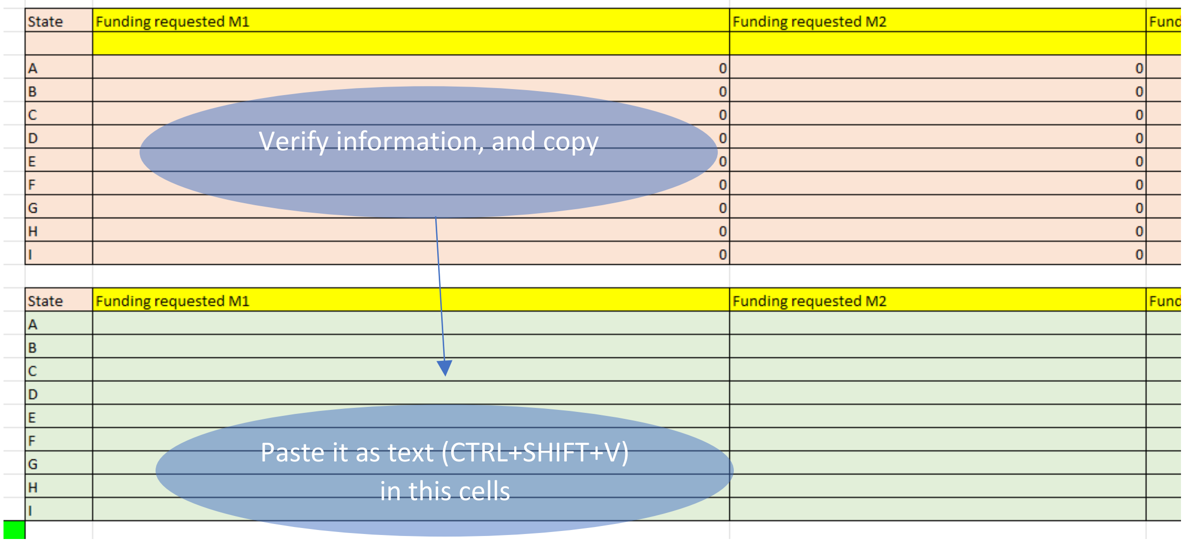

Figure 8 Step 1.2 Feedback Sheet

With this, we have completed Step 1 of the facilitator. In the figure below, the step we just completed is shown in orange.

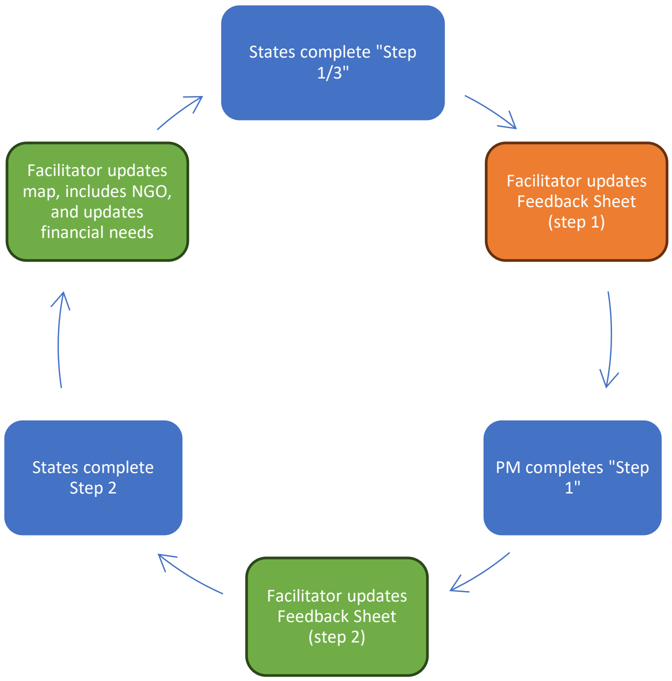

Now, information is displaying in the PM Sheet. Once the PM allocates funds to students (their Step 1), we follow with the Step 2 of the facilitator.

Step 2 for Feedback Sheet

Copy the values from the orange cell to the green cells (see figure 9). Verify that numbers are typed correctly (e.g., 10) with no text.

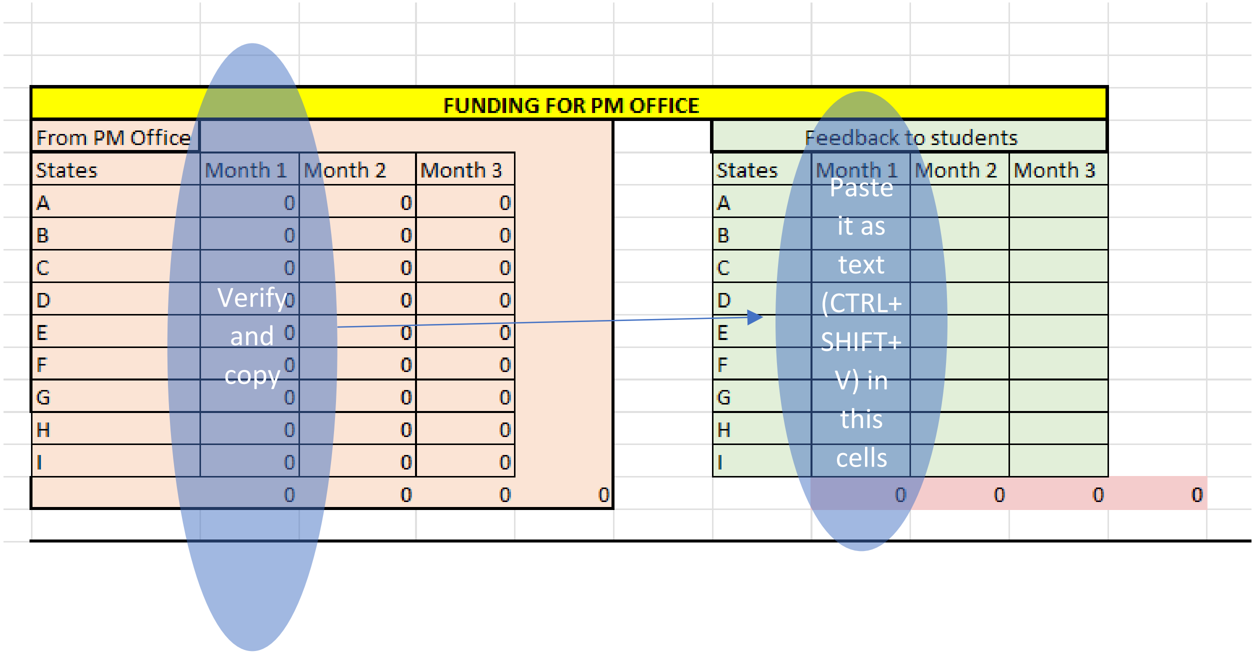

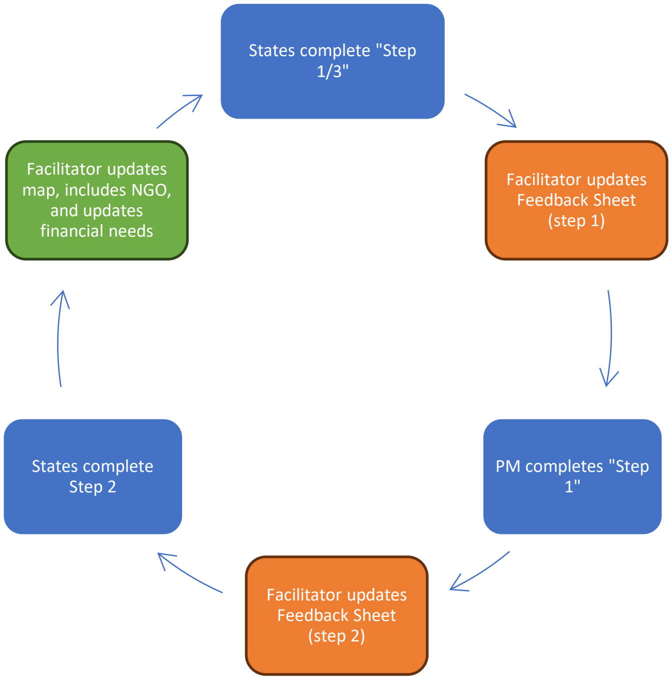

Step 3 for Feedback Sheet

Now that the states have allocated fund and personnel, we must complete three steps.

Step 3.1 Include data from NGOs.

The NGO group does not have access to the Excel file. When they decide which group they are helping with personnel and funding, they must let the facilitator know. The NGO must tell them what the funds and personnel are for (e.g., funds are for State A, to invest in Bednets). With this information, we open the Master Sheet in the Excel.

In column S, for each state, we see their particular options for interventions (see figure below). Once the NGO communicates which intervention they are helping address, we find it in the list. For our example above, we highlight bednets.

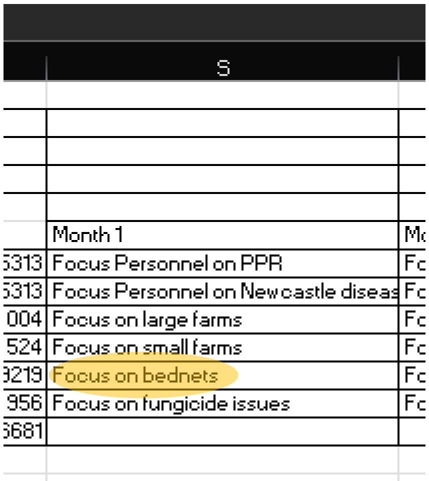

Figure 9 Step 3.1.1

After Identifying the row in which bednets are (11 in our example), we go to columns D and E (for month 1, for Month 2 it's columns I and J, and Month 3 are N and O). For easy access, they are shown in yellow.

In Cell D11 (based on our example) we type the number 1 (just as: 1). This is a binary variable to indicate there are personnel in this intervention. For cell E11, we type the number of millions the NGO is providing for this intervention. This amount is 5, based on the scenarios provided. If the scenarios are modified, then this amount may be different.

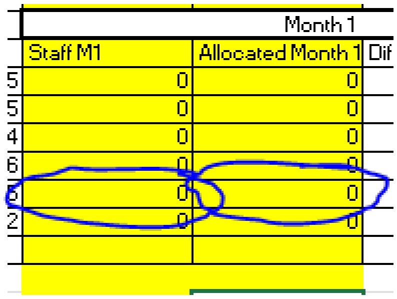

Figure 10 Step 3.1.2

<u>IMPORTANT:</u> Doing these steps above overrides formulas. That is ok, each excel sheet is only meant to be used once. If the instructors wish to repeat the simulation with another class, they should not recycle files, and they should start with a new one. Also, **BE CAREFUL** when identifying the cells targeted for change. The example above uses State A as an example, but the rows will be different for other states. For instance, for state B, the cells that target health interventions are rows 26 and 27. Also, states have different problems, which is why it is important to double-check the correct row number in Row S.

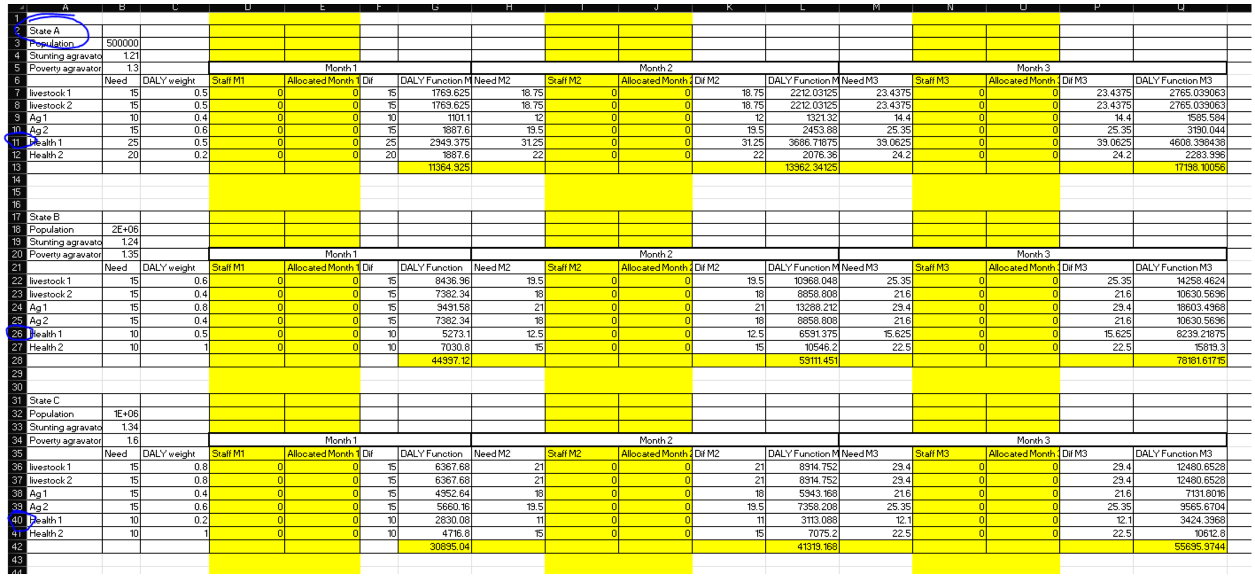

	A	B	C	D	E	F	G	H	I	J	K	L	M	N	O	P	Q
1																	
2	State A																
3	Population	500000															
4	Stunting agravato	1.21															
5	Poverty agravator	1.3		Month 1				Month 2					Month 3				
6		Need	DALY weight	Staff M1	Allocated Month 1	Dif	DALY Function M	Need M2	Staff M2	Allocated Month 2	Dif M2	DALY Function M	Need M3	Staff M3	Allocated Month 3	Dif M3	DALY Function M3
7	livestock 1	15	0.5	0	0	15	1769.625	18.75	0	0	18.75	2212.03125	23.4375	0	0	23.4375	2765.039063
8	livestock 2	15	0.5	0	0	15	1769.625	18.75	0	0	18.75	2212.03125	23.4375	0	0	23.4375	2765.039063
9	Ag 1	10	0.4	0	0	10	1101.1	12	0	0	12	1321.32	14.4	0	0	14.4	1585.584
10	Ag 2	15	0.6	0	0	15	1887.6	19.5	0	0	19.5	2453.88	25.35	0	0	25.35	3190.044
11	Health 1	25	0.5	0	0	25	2949.375	31.25	0	0	31.25	3686.71875	39.0625	0	0	39.0625	4608.398438
12	Health 2	20	0.2	0	0	20	1887.6	22	0	0	22	2076.36	24.2	0	0	24.2	2283.996
13							11364.925					13962.34125					17198.10056
14																	
15																	
16																	
17	State B																
18	Population	2E+06															
19	Stunting agravato	1.24															
20	Poverty agravator	1.35		Month 1				Month 2					Month 3				
21		Need	DALY weight	Staff M1	Allocated Month 1	Dif	DALY Function	Need M2	Staff M2	Allocated Month 2	Dif M2	DALY Function M	Need M3	Staff M3	Allocated Month 3	Dif M3	DALY Function M3
22	livestock 1	15	0.6	0	0	15	8436.96	19.5	0	0	19.5	10968.048	25.35	0	0	25.35	14258.4624
23	livestock 2	15	0.4	0	0	15	7382.34	18	0	0	18	8858.808	21.6	0	0	21.6	10630.5696
24	Ag 1	15	0.8	0	0	15	9491.58	21	0	0	21	13288.212	29.4	0	0	29.4	18603.4968
25	Ag 2	15	0.4	0	0	15	7382.34	18	0	0	18	8858.808	21.6	0	0	21.6	10630.5696
26	Health 1	10	0.5	0	0	10	5273.1	12.5	0	0	12.5	6591.375	15.625	0	0	15.625	8239.21875
27	Health 2	10	1	0	0	10	7030.8	15	0	0	15	10546.2	22.5	0	0	22.5	15819.3
28							44997.12					59111.451					78181.61715
29																	
30																	
31	State C																
32	Population	1E+06															
33	Stunting agravato	1.34															
34	Poverty agravator	1.6		Month 1				Month 2					Month 3				
35		Need	DALY weight	Staff M1	Allocated Month 1	Dif	DALY Function	Need M2	Staff M2	Allocated Month 2	Dif M2	DALY Function M	Need M3	Staff M3	Allocated Month 3	Dif M3	DALY Function M3
36	livestock 1	15	0.8	0	0	15	6367.68	21	0	0	21	8914.752	29.4	0	0	29.4	12480.6528
37	livestock 2	15	0.8	0	0	15	6367.68	21	0	0	21	8914.752	29.4	0	0	29.4	12480.6528
38	Ag 1	15	0.4	0	0	15	4952.64	18	0	0	18	5943.168	21.6	0	0	21.6	7131.8016
39	Ag 2	15	0.6	0	0	15	5660.16	19.5	0	0	19.5	7358.208	25.35	0	0	25.35	9565.6704
40	Health 1	10	0.2	0	0	10	2830.08	11	0	0	11	3113.088	12.1	0	0	12.1	3424.3968
41	Health 2	10	1	0	0	10	4716.8	15	0	0	15	7075.2	22.5	0	0	22.5	10612.8
42							30895.04					41319.168					55695.9744
43																	

Figure 11 Step 3 Clarifying Figure

Step 3.2 Update Map

Copy the DALYs from the orange cells labeled DALYs from states from the "Feedback sheet" (see image below) and **paste them as text** in the green cells labeled DALYs for students (see figure 12). This will automatically update the Interactive Map, and will also provide feedback to the students about their DALY's per sector

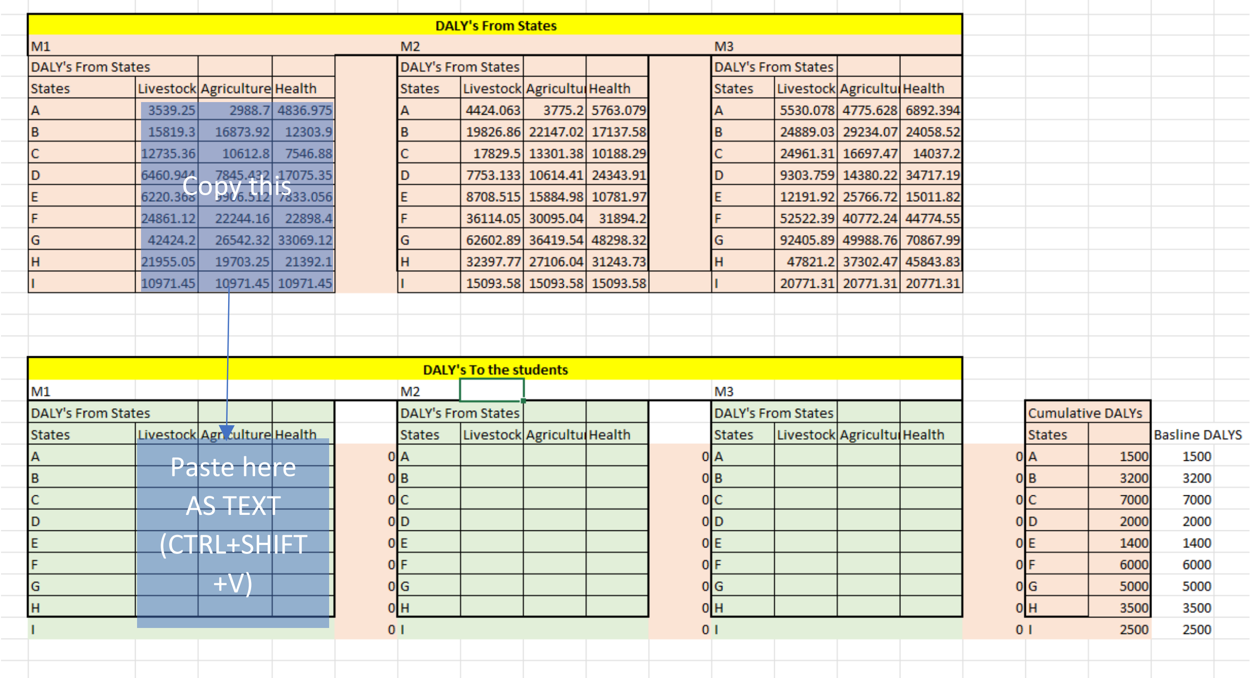

DALY's From States

M1					M2					M3			
DALY's From States					DALY's From States					DALY's From States			
States	Livestock	Agriculture	Health		States	Livestock	Agricultu	Health		States	Livestock	Agricultu	Health
A	3539.25	2988.7	4836.975		A	4424.063	3775.2	5763.079		A	5530.078	4775.628	6892.394
B	15819.3	16873.92	12303.9		B	19826.86	22147.02	17137.58		B	24889.03	29234.07	24058.52
C	12735.36	10612.8	7546.88		C	17829.5	13301.38	10188.29		C	24961.31	16697.47	14037.2
D	6460.94[illegible]	7845.4[illegible]2	17075.35		D	7753.133	10614.41	24343.91		D	9303.759	14380.22	34717.19
E	6220.36[illegible]	[illegible]906.512	7833.056		E	8708.515	15884.98	10781.97		E	12191.92	25766.72	15011.82
F	24861.12	22244.16	22898.4		F	36114.05	30095.04	31894.2		F	52522.39	40772.24	44774.55
G	42424.2	26542.32	33069.12		G	62602.89	36419.54	48298.32		G	92405.89	49988.76	70867.99
H	21955.05	19703.25	21392.1		H	32397.77	27106.04	31243.73		H	47821.2	37302.47	45843.83
I	10971.45	10971.45	10971.45		I	15093.58	15093.58	15093.58		I	20771.31	20771.31	20771.31

DALY's To the students

M1					M2					M3							
DALY's From States					DALY's From States					DALY's From States					Cumulative DALYs		
States	Livestock	Agriculture	Health		States	Livestock	Agricultu	Health		States	Livestock	Agricultu	Health		States		Basline DALYS
A				0	A				0	A				0	A	1500	1500
B				0	B				0	B				0	B	3200	3200
C				0	C				0	C				0	C	7000	7000
D				0	D				0	D				0	D	2000	2000
E				0	E				0	E				0	E	1400	1400
F				0	F				0	F				0	F	6000	6000
G				0	G				0	G				0	G	5000	5000
H				0	H				0	H				0	H	3500	3500
I				0	I				0	I				0	I	2500	2500

Step 3.2 Update State's Financial Needs

Copy the financial needs from the orange cells labeled "Financial Needs" from states from the "Feedback sheet" (see image below) and **paste them as text** in the green cells labeled Financial Needs to students (see figure 13). This will automatically update the students financial needs in their state's sheet. State I is in Dark Green as a way to facilitate visually when copying.

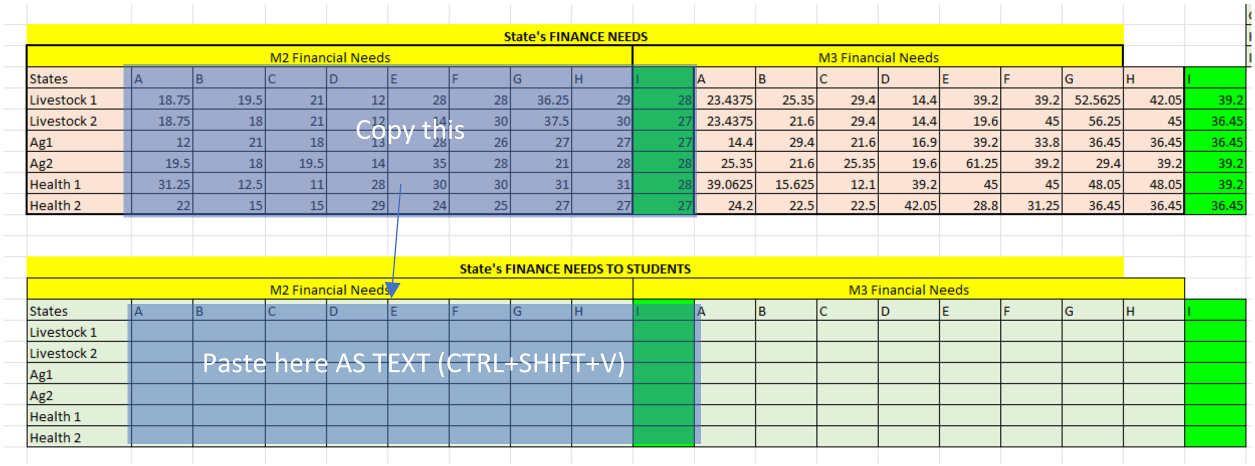

State's FINANCE NEEDS

States	M2 Financial Needs A	B	C	D	E	F	G	H	I	M3 Financial Needs A	B	C	D	E	F	G	H	I
Livestock 1	18.75	19.5	21	12	28	28	36.25	29	28	23.4375	25.35	29.4	14.4	39.2	39.2	52.5625	42.05	39.2
Livestock 2	18.75	18	21	12	14	30	37.5	30	27	23.4375	21.6	29.4	14.4	19.6	45	56.25	45	36.45
Ag1	12	21	18	13	28	26	27	27	27	14.4	29.4	21.6	16.9	39.2	33.8	36.45	36.45	36.45
Ag2	19.5	18	19.5	14	35	28	21	28	28	25.35	21.6	25.35	19.6	61.25	39.2	29.4	39.2	39.2
Health 1	31.25	12.5	11	28	30	30	31	31	28	39.0625	15.625	12.1	39.2	45	45	48.05	48.05	39.2
Health 2	22	15	15	29	24	25	27	27	27	24.2	22.5	22.5	42.05	28.8	31.25	36.45	36.45	36.45

State's FINANCE NEEDS TO STUDENTS

States	M2 Financial Needs A	B	C	D	E	F	G	H	I	M3 Financial Needs A	B	C	D	E	F	G	H	I
Livestock 1																		
Livestock 2																		
Ag1																		
Ag2																		
Health 1																		
Health 2																		

With this, you have finished the first cycle of the simulation. Repeat for months 2 and 3, but copying and pasting values for the corresponding cells. The above screenshots are only applicable for Month 1, for Month 2, and 3, the steps are the same, just in different columns that are labeled accordingly.

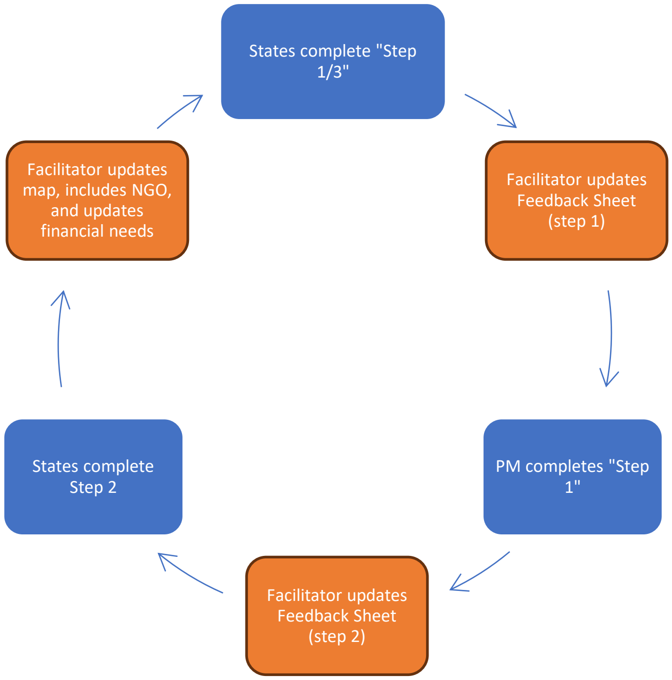

Info on the Master Sheet

Except for step 3.1.1, there is no need to modify this sheet. This is the sheet where all calculations are done. If there are questions, or an instructor wishes to modify the back end of this sheet, please reach out to the corresponding author: daniel.acosta@ufl.edu or danieacostag@gmail.com

Interactive Map

The interactive map will update itself by following the steps outlined above. The map is programmed to shade green those with lower DALY's and go progressively to Red to those with higher DALYs, with yellow as a midpoint. This can visually help each group understand how each state stands in comparison to other states. It also shows those states that are more content. The average discontent level is also shown. As a reminder, if the discontent level reaches 3.8 (instructors are free to change this number), then the initial budget will be reduced 200 million. This reduction has to be done manually by the facilitator, in the sheet called "Office of the PM" cell N3.

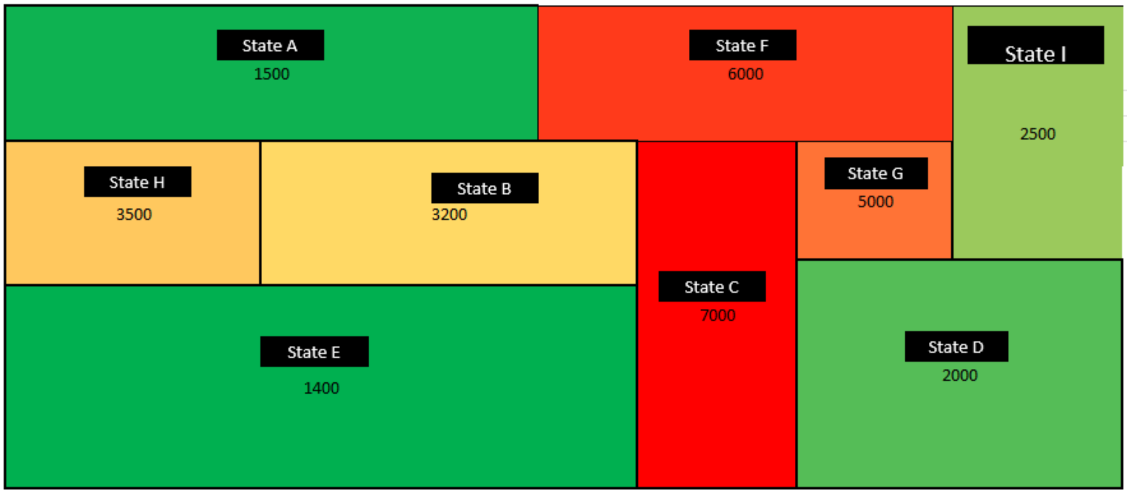

Initial Budget	$	700.00
Total Budget Availabl	$	700.00
Total expenditure	$	-

Happiness Level	
State A	0
State B	0
State C	0
State D	0
State E	0
State F	0
State G	0
State H	0
State I	0

Discontent Level
#DIV/0!

Carrying out the Simulation

There are several mechanisms and checks in place to ensure that the simulation runs smoothly. Once the simulation starts, the facilitator managing the Google Sheets will oversee keeping the simulation running, as outlined above. We recommend 3 hours to carry out the simulation; we advise against trying to carry out the simulation in less than 2 hours of time. At the end of Month 2, the stakeholder meeting should take place. A complete diagram of the flow of the simulation is shown below. Month 1 is in blue, Month 2 is in orange, and Month 3 is in Green. Facilitator steps (those to ensure the smooth flow of the simulation, that do not involve students, are shown in gray). **<u>We strongly recommend piloting the activity with a small group before carrying it out in the class setting.</u>**

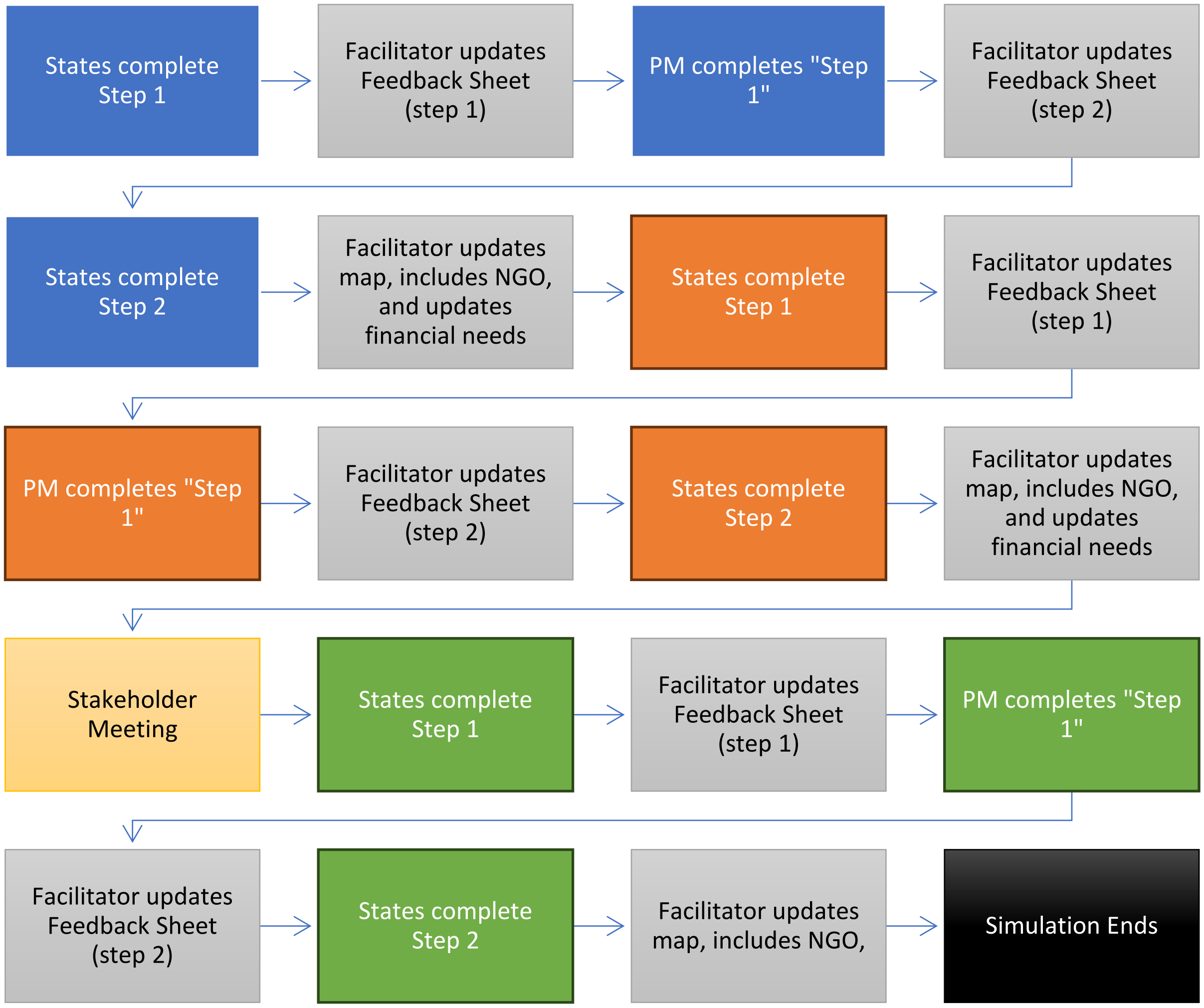

Timing

We recommend **<u>at least 10 minutes</u>** between steps (not including Facilitator Steps, those should be done immediately to avoid delays). The facilitator is in charge of keeping time. The stakeholder meeting is recommended for at least 15 minutes. We also recommend building in time to explain to students how to interact with the Excel file. This is why we recommend a 3-hour period for the activity.

Stakeholder Meeting

For the stakeholder meeting, students separate from their group and meet disciplinarily. All of the Health officers meet with the Ministry of Health, all of the Agricultural officers meet with the ministry of Agriculture, all of the Veterinary officers meet with the Ministry of Livestock, and all of the mayors meet with the Prime Ministers. NGOs are welcome to come to whatever meeting they choose. These simultaneous meetings should last between 15-20 minutes. The objective of these meetings is for states to make their case to actors at the national level.

Context and Details of each State

Below are the handouts to students of each group, where the scenarios are outlined.

Prime Minister's Office

This is the highest level of government and have had a relatively good tenure. You are up for re-election soon, and you need to win. As Prime Minister, you oversee 17 different ministries, and under the guidance of congress, allocates their respective budgets. The current emergency response fund for health, livestock, and agriculture is $800 million USD for the next 3 months. Most of these funds come from international donors, so maintaining the country stable is very important to ensure access to these funds. If the country reaches an average of >3.8 in terms of discontent, the risk of a coup d'état increases, leading to a reduction in donor support (total country budget gets cut $200 Million, and INGO will cease all operations in the country). This will also mean violent conflict that could lead to the deaths of many.

As Prime Minister, there are important issues to consider when it comes to prioritizing resources. These are important key points to remember:

- Coffee, sugar, and tea exports are an important part of the country's GDP. Issues with coffee production could have negative economic effects in the country for years to come.
- Farm-based cattle production is used for export, which is also an important part of the GDP.
- Pastoral and agropastoral production of livestock is mainly used for local consumption. Failures in livestock production could increase prices of food products and create civil unrest.
- Sorghum and maize production are key to food security in the country.
- High levels of mortality from infectious diseases detracts tourism (cholera, dengue, malaria). The president has promised the country that they will increase tourism revenue.
- People from states C, F, and G are mostly from an ethnic minority. There have been accusations from international donors that the government of the *Republic of Gators* is not taking the concerns of these populations as seriously as the ones from the majority ethnic group. If these trend continues, external support might reduce.

Some additional information that is confidential, but you have decided to share with your trusted **Ministers.**

- Your cousin in Mayor of State A, but there is not a love between you two. You need the tax revenue from State A, and keeping them happy is important.
- The Mayor of State B is a very good friend. You don't think they will be mad if funds don't flow their way in an emergency.
- The Mayor of State C is very corrupt, BUT, you need them for their influence and their incredible ability to secure votes.
- The Mayor of State D is your sibling, this is no secret. What some do not know if that your nephew hates you and is trying to convince your sibling to foment discontent.
- The Mayor of State E is a very good friend, and very wealthy. You hope they understand if you can't fund them properly.
- The Mayor of State F is your political rival, but, you both understand the importance of this state, so you try not to play politics.
- You don't know the new Mayor of Stage G, but historically, this has been a very corrupt state. You have heard this new Mayor is different, but that remains to be seen.

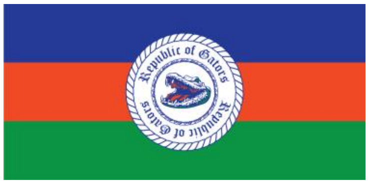

- In private conversations, the Mayor of State H has told you that keeping discontent at bay has been hard in this state. This state is highly vulnerable, and a lot of investment is needed.
- It is not a mystery that there is no love between you and the Mayor of State I, however, you are not enemies either. They are a very hard person to read.

First task: Decide how much to allocate for the first month each state. Remember the scenario is three months long, and you can allocate resources 2 more times.

As Prime Minister, you decide what the priorities are, but remember use your trusted advisors (Minister of Agriculture, Minister of Livestock, and Minister of Health). Each state will manage the funds they receive as best as they can. Sometimes interventions would work, sometimes they would fail. The fail/success rate is directly correlated to the amount invested.

State A: Context

Month 1-3

Below is the situation of your state for the following 3 months. If you address an issue (partially or fully), then the next month the issue might be resolved, or its impact minimized. The needs for the second and third month will vary depending on the decisions made in previous months. Remember that INGO could help, both financially and with personnel. Remember that you can change the level of discontent of your state. If the country reaches an average of >3.8 in terms of discontent, the risk of a coup d'état increases, leading to a reduction in donor support (total country budget gets cut $200 Million, and INGO will cease all operations in the country).

Mayor

You got elected in great part because of the support of the coffee associations. Not only did they support you, but they are also the main economic activity in the state. They generate most of the employment and are one of the main sources of tax revenue. It's important to maintain this sector happy, especially because they will help you get re-elected. Things to consider:

- Coffee requires intensive labor; a healthy population is as important as healthy crops.
- The Prime Minister is your cousin, but your relationship is not that good. Normally, you support their campaign by convincing the coffee associations to vote and financially support them. You are thinking about lightly threatening the PM if you don't receive the full amount of funds you request.
- Your state is fairly wealthy and not suffering from high rates of stunting or poverty. You would not be very affected in the long-term if the discontent level reaches a critical point for the country. Don't be afraid to raise civil unrest if needed.

Senior Veterinary Officer

You are very worried right now because your junior officers keep finding symptoms of peste des petite ruminants (PPR) in goats and sheep across all the state. Livestock is not very important commercially, but it is the main source of nutrient dense food of the population. A PPR outbreak would be devastating to the local community and could lead to hunger and unrest across the state. You need more funds to test for PPR, because positive PPR results would help you obtain vaccines. Ideally, you would need $15 Million USD to conduct a statewide diagnostic and vaccination campaign. To make matter worse, there are some concerns with poultry dying in some farms. You suspect it could be Newcastle disease (NCD) which would cost $15Million USD to diagnose and control with vaccines. This is very worrisome because a NCD outbreak could completely wipe out the poultry population of your state, leading to food insecurity for households who depend on chicken and eggs as part of their livelihood. You only have enough veterinarians in your staff to carry out one testing and vaccination activity, so you must choose to either focus on PPR or in NCD.

Senior Agricultural Officer

You just arrived from a meeting with the coffee associations and there are important concerns about an imminent outbreak of Coffee Berry Disease, a fungal pathogen that would affect quality of coffee beans, a huge threat to exports. It is urgent to implement fungicide treatment to all coffee in the state. Failing

to do so would be very risky. There are two groups of farmers in the state. Those who are very wealthy and own large coffee farms, and those who own small farms, who make small profits and are often food insecure. You don't have enough personnel to tackle both large and small farms. Treating the large farms would keep the wealthy farmers and their employees happy, but might trigger food insecurity across the region if small farms fail. The cost of treatment for the large farms is $10 Million. The cost of treatment of small farms is $15 million (as you need more motorcycles and gasoline to reach these areas).

Senior Health Officer A

Malaria is on the rise again as the rainy season is starting. There needs to be a campaign to distribute and promote the use of bednets. The cost of bednets and promotional campaign is $25Million. You also heard the Senior Agricultural Officer talk about fungicide applications, which makes you worried, as usually clinics see an uptick of fungicide exposure illnesses, adding stress to your already stretched out clinics. The clinics would need about $20 Million to do outreach to treat fungicide issues, as they need to reach farms quickly to ensure coffee production doesn't stop. You only have enough personnel to carry out a bednet campaign and to prepare the clinics for an influx of fungicide intoxications at the same time, so you must choose on which to focus the resources that will be provided.

State B

Month 1-3

Below is the situation of your state for the following 3 months. If you address an issue (partially or fully), then the next month the issue might be resolved, or its impact minimized. The needs for the second and third month will vary depending on the decisions made in previous months. Remember that INGO could help, both financially and with personnel. Remember that you can change the level of discontent of your state. If the country reaches an average of >3.8 in terms of discontent, the risk of a coup d'état increases, leading to a reduction in donor support (total country budget gets cut $200 Million, and INGO will cease all operations in the country).

Mayor

You got elected in great part because of the support of the goat associations. They might not be the biggest economic activity of the state, but they are certainly an important part of the state's economy. The sugar associations did not fully support you at first, but they are the main economic activity in the state, and so far you have gained favor with their leaders.

- Sugar requires intensive labor, a healthy population is as important as healthy crops
- Goats in this state are a close second to sugar in terms of economic activity and first in terms of food security.
- You really don't want to anger the goat or sugar associations. Doing so could end your tenure as major.
- The Prime Minister is a good friend of yours. Hopefully he remembers that when allocating funds to you. Your friendship is solid, you understand if not enough money comes to your state. You would not raise the level of discontent, unless you feel the PM has been very very unfair…

Senior Veterinary Officer

You are very worried right now because your junior officers keep finding symptoms of peste des petite ruminants (PPR) in goats and sheep across all the state, as well as Foot and Mouth Disease (FMD) in pigs. Livestock is somewhat important commercially and it is the main source of nutrient dense food of the population. A PPR and FMD outbreak would be devastating to the local community. You only have enough personnel to carry out epidemiological surveillance and control for one of the diseases. You will need to choose which issue to address; PPR on goats and sheep or for FMD in pigs. Ideally, you would need $15 Million USD to conduct a statewide diagnostic for either disease. The closer to $15M, the better.

Senior Agricultural Officer

You just arrived from a meeting with the sugar associations and there are important concerns about several pests affecting sugarcane such as root borer. If true, the state would need a lot of pesticide quickly as currently there is not much available in the area. It is urgent to carry out several field trips to determine the level of risk. The diagnosis trips and pesticide would cost around $15 million USD. The

sugar association takes it for granted that investments will flow their way, after all, they are very powerful in the state. Another potential issue that has been brought to your attention is that maize plantations are facing challenges with fungal infections. Maize is used to feed livestock. You need $15 Million USD to take care of this. Not doing this could have important ramifications on food security, Furthermore, prices of feed will skyrocket, and the Goat associations will not be happy about that. You only have enough personnel to either treat the sugarcane or treat the maize.

Senior Health Officer

Malaria is on the rise again as the rainy season is starting, but you are well prepared with bednets donated by an NGO. It would be very good to carry out a campaign promoting the correct use of bednets, this would cost around $10 million USD. This, however, is the least of your problems. Some of your junior officers in the rural areas suspect of a cholera outbreak, with 3 deaths reported so far. You need $10 million dollars to react to this. An unchecked cholera outbreak could be devastating to the state if left unchecked. You only have enough personnel to either focus on Malaria, or in the suspected cholera outbreak. You are worried, as both of these issues are very important, however, you suspect that most funds will either be given interventions that benefit the sugar and goat associations.

State C

Month 1-3

Below is the situation of your state for the following 3 months. If you address an issue (partially or fully), then the next month the issue might be resolved, or its impact minimized. The needs for the second and third month will vary depending on the decisions made in previous months. Remember that INGO could help, both financially and with personnel. Remember that you can change the level of discontent of your state. If the country reaches an average of >3.8 in terms of discontent, the risk of a coup d'état increases, leading to a reduction in donor support (total country budget gets cut $200 Million, and INGO will cease all operations in the country).

Mayor

This is the poorest state of the country, but you run a tight ship and have popular support across the state. While Maize is the most important economic activity, chickens and eggs are very important for subsistence, as they are the main source of protein in the diets. Rates of infectious diseases is always high. You were elected under the promise that you would lift the state out of poverty, and while that has not happened maize prices keep going up in the national market, so everyone in the Maize business (including you, who owns one of the largest maize farms) is doing better. You have been accused of corruption before, but you think these allegations are false. It is entirely by coincidence that most agricultural interventions target your farms first. People at the national level are just jealous of your popularity in this large state.

Senior Veterinary Officer

You are very worried right now because your junior officers keep reporting massive deaths in chickens across all the state. You suspect is Newcastle Disease (NCD). Chickens are the main source of nutrient dense food of the population. A NCD outbreak would be devastating to the local community. You need $15 Million to address this issue. There are also talks about a contagious disease in goats, which if is peste des petite ruminants (PPR) or foot and mouth disease (FMD), could mean serious trouble as well. You also need $15 Million USD to address this problem. You only have enough personnel to do one of the two interventions (either chickens or goats).

Senior Agricultural Officer

Maize plants across the entire state are showing signs of phosphorus deficiency. A good and easy way to address this issue is to use manure as fertilizer, however, you need an educational campaign with farmers to do this. Your cousin (the mayor, whom you owe your job to) is insisting that you try this out in his farms first. He says it's to make sure this works and not risk the people's farms. You both know this would work, and this is just yet another excuse to get preferential treatment. This intervention would cost around 15 million USD. There is also some suspicion about a highly contagious fungal diseases in maize in the western part of the state, where most of the small producers live. To investigate, and control (if it's indeed a fungal disease) would cost $15 Million USD. You only have enough personnel to do one of the two interventions.

Senior Health Officer

Malaria is on the rise again as the rainy season is starting, but you are well prepared with bednets donated by an NGO. It would be very good to carry out a campaign promoting the correct use of bednets, this would cost around $10 million USD. This, however, is not your only problem. One of your junior officers in the rural areas suspect of a cholera outbreak, with 3 deaths reported so far. You need $10 million dollars to react to this, but only have enough personnel to focus on one of the interventions. An unchecked cholera outbreak could be devastating to the state if left unchecked. You only have enough personnel to either focus on Malaria, or in the suspected cholera outbreak. You are worried, as both of these issues are very important, however, you suspect that most funds will end up in the Mayor's farms, as usual.

State D

<u>Month 1-3</u>

Below is the situation of your state for the following 3 months. If you address an issue (partially or fully), then the next month the issue might be resolved, or its impact minimized. The needs for the second and third month will vary depending on the decisions made in previous months. Remember that INGO could help, both financially and with personnel. Remember that you can change the level of discontent of your state. If the country reaches an average of >3.8 in terms of discontent, the risk of a coup d'état increases, leading to a reduction in donor support (total country budget gets cut $200 Million, and INGO will cease all operations in the country).

Mayor

You and your family own one of the largest cattle farms for export. Your state is one of the most prosperous. The Prime Minister is your sibling, and while you have had your differences, you have also become very wealthy since they took power. You hold a lot of power in the region though, as your states cattle herd is a significant portion of the country's GDP. There is, however, issues of inequality, especially with crop producers which don't make as much as cattle farmers. However, crop producers are key to your livestock business (as you need their products for feed). You expect that any monetary request you make to the Prime Minister's Office will be met, after all, you are siblings. Your son, the senior veterinary officer of the state has no love for the prime minister, as he says you could run on the next election (or fund a coup) and take the position. But that's just because he is very young and ambitious, there is no need to raise the level of discontent if money keeps flowing your way that is…

Senior Veterinary Officer

You are in a constant state of vigilance, as an outbreak in cattle could have severe impacts on livestock production and hit the economy hard. 60% of the livestock population are cattle, 25% are goats, and 15% are chickens. You have enough veterinarians in your staff to focus on either cattle or goats and chicken in terms of epidemiologic surveillance. Most of the time you focus on cattle, so often goats and chickens are underserved. You need 10 million dollars to carry out diagnostic surveillance and address any threat, but due to personnel constraints, you can either do cattle, or chicken and goats. If you choose to surveille chicken and goats, you will lose surveillance in cattle. You would not have these issues if you parent was Prime Minister…

Senior Agricultural Officer

Feed production is on decline. Maize crops keep faltering in yield, and it seems to be a fungal disease, which needs a very strong fungicide. You need 10 million to carry out preventive measures for fungal diseases. There is also a growing concern in terms of sorghum production, which is mostly low scale done by women in their gardens. You would need to carry out some field visits to see what the issue is, to buy proper supplies. You only have personnel to carry out one of these interventions. It would cost $10 Million as well.

Senior Health Officer A

Malaria is on the rise again as the rainy season is starting, and you are very concerned as children from families that focus on crop production are showing signs of malnutrition. This is very dangerous, as malnourished children during Malaria season might lead to increased mortality. You have two options, do a bednet campaign with those communities (cost $20M) or do a nutritional intervention (cost $20M). You can't do both as you are short on personnel.

State E

Month 1-3

Below is the situation of your state for the following 3 months. If you address an issue (partially or fully), then the next month the issue might be resolved, or its impact minimized. The needs for the second and third month will vary depending on the decisions made in previous months. Remember that INGO could help, both financially and with personnel. Remember that you can change the level of discontent of your state. If the country reaches an average of >3.8 in terms of discontent, the risk of a coup d'état increases, leading to a reduction in donor support (total country budget gets cut $200 Million, and INGO will cease all operations in the country).

Mayor

You are the mayor of the richest state in the country thanks to the massive production of large-scale tea for export. You are also very wealthy, but your wealth does not come from tea, or agriculture, but from the numerous government contracts your construction company gets from the central government for the development of infrastructure. Needless to say, your relationship with the Prime Minister is quite good, and you have funded their campaign extensively in the past. Now that you are Mayor, you expect large support from the PM, after all, you were instrumental on getting them elected. In terms of the population of the state, other than the wealthy tea farmers, some farmers that produce other crops and products, which are important for your state's food security (meat, pork, goat meat, milk, chicken eggs). These farmers are also doing fairly well, however, those who produce staple foods only, such as sorghum and maize, struggle more and are generally under the poverty line.

Senior Veterinary Officer

Your state has low populations of livestock compared to other states, but a higher diversity in terms of the species you manage, and animals are spread out across the state. Even though you have limited personnel, you have managed to maintain a good disease surveillance system in place for cattle, chickens, goats, and pigs (although it's a little bit stretched out). You have heard some complaints of farmers in the north who say their young goats are dying (they describe symptoms of goat pox). Also in the north, chicken deaths have been reported. Your current surveillance system has not caught anything, but these increasing reports have you nervous. You could use about 20 million and move your veterinary officers in the south to the north to do a complete diagnostic and address any issues, but this means that the south (where pigs and cattle are) would be unmanned for a month, which could allow for the spread of diseases. Another option is maintaining the current system (not moving the veterinarians to the north), which would cost around $10 million.

Senior Agricultural Officer

You are currently dealing with a fungal outbreak in major tea plantations. Efforts have been relatively successful, but you need 20 million (and the current staff you have) to keep the outbreak at check. You only have 1 member of you staff working on the other crops of the region. She is concerned that yields in maize and sorghum keep falling and asks for you to provide her with support.

She needs 20 million and the staff currently working with tea producers to succeed. If you send her the staff from the tea plantations, the fungal outbreak could spread. You need to decide if you focus your staff on the tea plantations or if you move them to maize and sorghum farms.

Senior Health Officer A

Malaria is on the rise again as the rainy season is starting, and you are very concerned as children from families that focus on crop production are showing signs of malnutrition. This is very dangerous, as malnourished children during Malaria season might lead to increased mortality. You have two options, do a bednet campaign with those communities (cost $20M) or do a nutritional intervention (cost $20M). You can't do both as you are short on personnel.

State F

Month 1-3

Below is the situation of your state for the following 3 months. If you address an issue (partially or fully), then the next month the issue might be resolved, or its impact minimized. The needs for the second and third month will vary depending on the decisions made in previous months. Remember that INGO could help, both financially and with personnel. Remember that you can change the level of discontent of your state. If the country reaches an average of >3.8 in terms of discontent, the risk of a coup d'état increases, leading to a reduction in donor support (total country budget gets cut $200 Million, and INGO will cease all operations in the country).

Mayor

You are the mayor of one of the poorest states in the country, and incidentally, one of the most important states when it comes to food production for local consumption. You are one of the most important political rivals of the current Prime Minister, but you both understand the importance of investing in this state. Regardless of the challenges, you have kept political movements and talks of rebellion at bay, as investments have been quite good. If investments stop flowing at the appropriate amount, you are inclined to raise the level of discontent. People in the state are mostly subsistence farmers, with low market orientation, however, given the size of the population, the collective herd size and crop production that go into the market are quite significant. You have the highest rate of stunting in the country, and it is your mission to address this (you were elected on the promise to end child malnutrition).

Senior Veterinary Officer

Your state is one of the main producers of livestock, focusing on cattle, goats, and sheep. You have struggled to control diseases given the agro-pastoral nature of production. It is very challenging to carry out any diseases control or surveillance when farmers are moving with their cattle. Most of the farmer that migrate are men, and with them most of the cattle, goats and sheep (these are the livestock sold at the markets that are important for the country's food production). Focusing on the migrating livestock would cost $20Million USD. The livestock that remains in the towns is a small amount, but it is widely spread out. It is used for household consumption and women are in charge of it. Taking care of the livestock that remain behind would also cost $20 Million. You don't have enough personnel to do both approaches so you have to decide whether focus your effort on the men owned livestock (who are currently migrating in search of pasture), or focus on the livestock that remained behind, used for household consumption of children and woman.

Senior Agricultural Officer

Sorghum and maize yields are down. If yields in maize and sorghum keep falling, this could worsen food security. It seems to be a pest issue (wire worms). You have enough supplies to address the issue in the whole state, however, only enough personnel to either focus on sorghum or maize. Sorghum is mostly used for animal feed, and addressing this issue would cost $20 Million USD. Maize is used for household consumption, and addressing issues with maize would cost $20 Million.

Senior Health Officer A

Malnutrition is prevalent in the whole state and with the rainy season coming. This is not good as the risk of malaria sharply increases and malnourished children are at highest risk of death. You need to decide whether you spend your efforts controlling for malaria (bednet campaign, cost $20 Million) or if you launch a nutritional intervention (food distribution + behavior change interventions to increase animal food source consumption, cost $20 Million).

State G

Month 1-3

Below is the situation of your state for the following 3 months. If you address an issue (partially or fully), then the next month the issue might be resolved, or its impact minimized. The needs for the second and third month will vary depending on the decisions made in previous months. Remember that INGO could help, both financially and with personnel. Remember that you can change the level of discontent of your state. If the country reaches an average of >3.8 in terms of discontent, the risk of a coup d'état increases, leading to a reduction in donor support (total country budget gets cut $200 Million, and INGO will cease all operations in the country).

Mayor

You are the mayor of one of the poorest states in the country, and incidentally, one of the most important states when it comes to food production for local consumption. There are high levels of inequality in your state, and there have been scandals of corruption in the past. You were elected on the promise that you will address these issues, but it has been challenging. You are neutral in terms of politics, so you have no love or hate for the current Prime Minister, but you do need a lot of funds if you are to address the issues of the state. You understand that raising discontent to 3.8 could hurt the country a lot, but if things don't improve, you don't know if you have a choice to stand idle. People in the state are mostly subsistence farmers, with low market orientation, however, given the size of the population, the collective crop production that go into the market are quite significant. You have one of the highest rates of stunting in the country, and it is your mission to address this.

Senior Veterinary Officer

Your state does not have a large heard, and most of the livestock is for self-consumption. Most of the livestock are goats, sheep, and chickens. You have struggled to control diseases given that the population is very spread out. It is very challenging to carry out any diseases control or surveillance as you are short staffed, and even if your veterinarians manage to cover the area, they can only really focus on either small ruminants (goats and sheep) or chickens. You have a perfect storm in your hands right now, as there are confirmed reports of both peste des petite ruminants (PPR, affects goats and sheep) and Newcastle disease (affects chickens). Both of these diseases could wipe out goats and chickens in the state. The cost of addressing either issue is $25 million USD. You only have enough veterinarians to address one of the issues.

Senior Agricultural Officer

Sorghum yields are down in the East and the West. If yields of sorghum keep falling, this could worsen food security. In the East, it seems to be a pest issue (wire worms), while in the West, it seems to be a fungal issue. You don't have enough personnel to address the issues in both the East and the West. Addressing wireworms' costs $20 Million and addressing the fungi problem costs $15 million.

Senior Health Officer A

Malnutrition is prevalent in the whole state and with the rainy season coming. This is not good as malaria cases on malnourished children could lead to high mortality. You need to decide whether you spend your efforts controlling for malaria (bednet campaign, cost $20 Million) or if you launch a nutritional intervention (food distribution + behavior change interventions to increase animal food source consumption, cost $20 Million).

State H

Month 1-3

Below is the situation of your state for the following 3 months. If you address an issue (partially or fully), then the next month the issue might be resolved, or its impact minimized. The needs for the second and third month will vary depending on the decisions made in previous months. Remember that INGO could help, both financially and with personnel. Remember that you can change the level of discontent of your state. If the country reaches an average of >3.8 in terms of discontent, the risk of a coup d'état increases, leading to a reduction in donor support (total country budget gets cut $200 Million, and INGO will cease all operations in the country).

Mayor

You are the mayor of one of the poorest states in the country, and one of the most important states when it comes to food production for local consumption. Your state has a large pastoral population, and the Prime Minister is not very popular with the population because the policies have mostly favored a farm-based production. Keeping the level of discontent at 1-2 would be hard if you don't get enough funds to properly address all of your issues. People in the state are mostly subsistence farmers, with low market orientation, however, given the size of the population, the collective herd size and crop production that go into the market are quite significant. You have one of the highest rates of stunting in the country, and it is your mission to address this (you were elected on the promise to end child malnutrition).

Senior Veterinary Officer

Your state is one of the main producers of livestock, focusing on cattle, goats, and sheep. You have struggled to control diseases given the pastoral nature of production. It is very challenging to carry out any diseases control or surveillance when farmers are moving with their cattle. Most of the farmer that migrate are men, and with them most of the cattle, goats and sheep (these are the livestock sold at the markets that are important for the country's food production). The livestock that remains in the towns is a small amount, used for household consumption, and women are in charge of it. You don't have any funds and you only have enough personnel to either focus your effort on the men owned livestock (who are currently migrating in search of pasture), or focusing on the livestock that remained behind, used for household consumption of children and woman. Either approach would cost $20Million, but you can only address one of the issues with the current personnel.

Senior Agricultural Officer

Sorghum and maize yields are down. These are both important products for local consumption of children and women, as well as feed for the cattle that remain behind. If yields in maize and sorghum keep falling, this could worsen food security. It seems to be a pest issue (wire worms). You have enough supplies to address the issue in the whole state, however, only enough personnel to either focus on sorghum or maize. Sorghum is mostly used for animal feed, while maize is used for household consumption. The campaign (for either crop) would cost $20 Million.

Senior Health Officer A

Malnutrition is prevalent in the whole state and with the rainy season coming. This is not good, as infectious diseases like Malaria have higher mortality in malnourished children. You need to decide whether you spend your efforts controlling for malaria (bednet campaign, cost $20 Million) or if you launch a nutritional intervention (food distribution + behavior change interventions to increase animal food source consumption, cost $20 Million). You only have enough personnel to carry out one of the interventions.

State I

Month 1-3

Below is the situation of your state for the following 3 months. If you address an issue (partially or fully), then the next month the issue might be resolved, or its impact minimized. The needs for the second and third month will vary depending on the decisions made in previous months. Remember that INGO could help, both financially and with personnel. Remember that you can change the level of discontent of your state. If the country reaches an average of >3.8 in terms of discontent, the risk of a coup d'état increases, leading to a reduction in donor support (total country budget gets cut $200 Million, and INGO will cease all operations in the country).

Mayor

Your state has always been doing relatively well when compared to your neighboring states F and G, but not as good as richer states. Your state has quite a diverse economy, which is why you think things are not as bad as in other states. People in the state are mostly farmers and livestock keepers, both for subsistence and for selling at the market. One of your main issues is the lack of governance at the national level, and in your opinion, the Prime Minister is weak, and you do not like them at all. Your family had some rivalry in the past with the family of the Prime Minister, which makes you even more likely to be discontent if your requests for funds are not met.

Senior Veterinary Officer

Your state has quite a mix of livestock, from poultry to cattle, but there are not many large scale farming operations. This makes surveillance a little bit tricky, and you rely heavily on your already overworked staff. Your staff has informed you of two confirmed outbreaks, one of peste des petits ruminants (affecting sheep and goats) and on of poultry (Newcastle disease). Just what you needed, two very deadly and highly contagious outbreaks at the same time… Thanks to your good planning (not the ministry for sure) you have vaccines for both diseases, which could help contain the outbreak. The problem is that you don't have sufficient staff, so you need to decide which vaccine to implement. Either way, the cost would be $20million.

Senior Agricultural Officer

All crop yields are down and farmers are really not happy about it. Right now, your main focus is maize and tea, which are the ones with more drastic issues. If yields in maize keep falling, this could threaten food security, but if tea yields keep falling, this could threaten the income of many farmers who rely solely on this cashcrop. You have enough supplies to address the issue in the whole state, however, only enough personnel to either focus on tea or maize. The campaign (for either crop) would cost $20 Million.

Senior Health Officer A

Malnutrition is prevalent in the whole state and with the rainy season coming. This is not good, as infectious diseases like Malaria have higher mortality in malnourished children. You need to decide whether you spend your efforts controlling for malaria (bednet campaign, cost $20 Million) or if you launch a nutritional intervention (food distribution + behavior change interventions to increase animal food source consumption, cost $20 Million). You only have enough personnel to carry out one of the interventions.

Country director

The priority of INGO headquarters for the *Republic of Gators* is to avoid famine. Knowing that food systems are very connected to health, agriculture, and livestock, you have been sent 2 teams of One Health practitioners for the next three months. These teams have the capacity to support a state in the areas of human health, livestock health, or crop protection. A team can only work in one issue per month, in one state at a time. Each team also has 5 million USD per month that they can bring to the state they are working on to support activities. As country director, you meet often consult with national level actors before making any decisions, so be sure to speak with the Prime Minister and your Regional Directors before making any decisions.

Regional Directors:

Each of you is in charge of one region, as shown below:

- Region 1
  - State A
  - State B
  - State C
- Region 2
  - State D
  - State E
  - State F
- Region 3
  - State G
  - State H
  - State I

Within your region, visit each state and see where the resources of INGO could be better spent. The final decision on how to deploy the One Health Teams lies with the Country Director.

Issues to consider:

- If civil unrest rises, you will be forced to leave the country. Hopefully it does not come to that.
- There are concerns of corruption among certain States (C, D, E, and H). You don't know if these concerns are warranted, however, be weary when deciding where to allocate resources. Make sure your Regional Directors talk to the majors of these states to make sure funds would be well spent.
