# Supplementary material for: The importance of incorporating systems thinking and One Health in global health classrooms: findings from a One Health simulation activity
Source: Front Public Health. 2024 Feb 28;12:1299116. doi: 10.3389/fpubh.2024.1299116 (PMC10933002; doi:10.3389/fpubh.2024.1299116)
Supplement: Supplementary file 3 [file Data_Sheet_2.DOCX]

**FGD-1 Guide**

1. Icebreaker: How did your group do in the *“One-Health Stakeholder Activity”*
2. What are your thoughts about the *One-Health Stakeholder Activity*?
3. From a learning perspective, what were the main advantages of the *One-Health Stakeholder Activity?*
4. What were drawbacks or flaws in the activity that you felt could impair the learning process?
5. Should this activity continue to be a part of the Global Health course, why or why not?
6. What were your 3 main takeaways from the activity?
7. Were there any other learning experiences not mentioned above that the activity helped you achieve?
8. Do you have any additional comments about the activity?
